# Glatiramer Acetate Immunomodulation: Evidence of Neuroprotection and Cognitive Preservation

**DOI:** 10.3390/cells11091578

**Published:** 2022-05-07

**Authors:** Arielle Kasindi, Dieu-Trang Fuchs, Yosef Koronyo, Altan Rentsendorj, Keith L. Black, Maya Koronyo-Hamaoui

**Affiliations:** 1 Department of Neurosurgery, Maxine Dunitz Neurosurgical Institute, Cedars-Sinai Medical Center, Los Angeles, CA 90048, USA; arielle.kasindi@cshs.org (A.K.); dieu-trang.fuchs@cshs.org (D.-T.F.); yosef.koronyo@cshs.org (Y.K.); altan.rentsendorj@cshs.org (A.R.); keith.black@cshs.org (K.L.B.); 2 Department of Biomedical Sciences, Cedars-Sinai Medical Center, Los Angeles, CA 90048, USA

**Keywords:** Copolymer-1 (Cop-1), glaucoma, Parkinson’s disease, Huntington’s disease, experimental autoimmune encephalomyelitis, AD, retinal inflammation, optic neuropathy, cerebral ischemia, neuropsychology

## Abstract

Novel, neuroprotective uses of Copaxone (generic name: glatiramer acetate—GA) are being examined, primarily in neurological conditions involving cognitive decline. GA is a well-studied synthetic copolymer that is FDA-approved for immune-based treatment of relapsing remitting multiple sclerosis (RRMS). Clinical studies have explored the potential mechanism of action (MOA) and outcomes of GA immunization in patients. Furthermore, results from these and animal studies suggest that GA has a direct immunomodulatory effect on adaptive and innate immune cell phenotypes and responses. These MOAs have been postulated to have a common neuroprotective impact in several neuroinflammatory and neurodegenerative diseases. Notably, several clinical studies report that the use of GA mitigated MS-associated cognitive decline. Its propensity to ameliorate neuro-proinflammatory and degenerative processes ignites increased interest in potential alternate uses such as in age-related macular degeneration (AMD), amyotrophic lateral sclerosis (ALS), and Alzheimer’s disease (AD). Preclinical studies are exploring less frequent subcutaneous administration of GA, such as once weekly or monthly or a single dosing regimen. Indeed, cognitive functions were found to be either preserved, reversed, or improved after the less frequent treatment regimens with GA in animal models of AD. In this systematic review, we examine the potential novel uses of GA across clinical and pre-clinical studies, with evidence for its beneficial impact on cognition. Future investigation in large-size, double-blind clinical trials is warranted to establish the impact of GA immunomodulation on neuroprotection and cognitive preservation in various neurological conditions.

## 1. Introduction

The synthetic immunoactive copolymer glatiramer acetate (GA; formula C_25_H_45_N_5_O_13_), branded Copaxone (also known as Copolymer-1 or Cop-1), is comprised of four amino acids in random order, resembling myelin basic protein (MBP) [1]. MBP is highly expressed during central nervous system (CNS) damage, specifically in autoimmune and/or inflammatory states such as multiple sclerosis (MS) or central degeneration [2]. GA was first synthesized in 1967 to induce experimental autoimmune encephalitis (EAE) in murine models of relapsing remitting multiple sclerosis (RRMS) [3]. Unexpectedly, GA was found to reduce signs and progression of EAE in these models [1,3]. Rather than inducing an autoimmune disease, GA was found to serve as a weak agonist to myelin-derived proteins and induce regulatory and protective neuroimmune responses [4,5]. Thus, GA was translated to clinical trials and was approved for use as an RRMS treatment in 1996 [6].

Multiple sclerosis (MS) is an inflammatory, demyelinating disease of the CNS with a prevalence of >1% in North America and Europe, and 0.002% in Eastern Asia and sub-Saharan Africa [7]. RRMS, a subtype of MS, accounts for around 85% of MS cases worldwide [7]. It is characterized by asymptomatic periods followed by a relapse or reoccurrence of symptoms [8].

Current treatments for RRMS include Interferon-β (IFN-β), S1P inhibitors such as fingolimod, monoclonal antibodies such as natalizumab, and anti-CD20 therapies (rituximab, ocrelizumab, ofatumumab) [5]. IFN-β is an immunomodulatory drug, and fingolimod also acts on the immune system by inhibiting peripheral lymphocytic egress [5]. Although IFN-β is immunomodulatory and a first-line RRMS therapy option, like GA, it has not been shown to enter the brain parenchyma or spinal cord and have a direct effect in the CNS. Instead, it is believed to express an indirect immunomodulatory effect in the CNS [9]. Natalizumab, a second-line agent, is a recombinant IgG4 monoclonal antibody which blocks the α4 subunit of integrin on leukocytes, preventing leukocytes from entering the CNS [5]. Fingolimod and Natalizumab are newer drugs often used as second-line agents to treat RRMS due to their extensive side-effect profiles. Despite this broad range of therapy options, both novel and established, GA remains a first-line immunomodulation therapy option for RRMS due to its effectiveness and generally low side-effect profile [7].

However, recent studies are beginning to examine the full scope of GA’s immunomodulatory effects as well as its potential to ameliorate various aspects of RRMS and other neurological diseases. Most notably, studies are exploring the potential for GA to protect from cognitive decline. This paper aims to provide a comprehensive review of studies investigating novel applications and uses of GA in various neuroinflammatory and neurodegenerative processes, including age-related macular degeneration (AMD), Alzheimer’s disease (AD), cerebral ischemia, amyotrophic lateral sclerosis (ALS), neuropsychological conditions, glaucoma, Parkinson’s disease (PD), and Huntington’s disease (HD), and its potential impact on cognition.

### 1.1. Mechanism of Action

Although GA remains an established agent for treating RRMS and its disease course [6], its exact mechanism of action is not fully understood. However, to have a clear understanding of its known and hypothesized roles in RRMS, it is important to understand the pathophysiology of the disease. Multiple sclerosis is an autoimmune disease in which CD4^+^ autoreactive T cells target myelin and mount an inflammatory response in central neurons causing demyelination which leads to neurological deficits [9]. Various immune cell lines and inflammatory mediators are implicated in the pathophysiology of the disease. These include multiple derivatives of T cells, B cells, antibodies/autoantibodies, monocytes, macrophages, cytokines, and resident CNS immune cells such as microglia [10].

The impact of GA on CNS tissues as well as on peripheral immune cells is under investigation, with new properties of this agent being discovered. It has been extensively shown that one principal mechanism of action of GA is on the adaptive immune response [11]. Specifically, as GA resembles MBP, it has been found to competitively and antagonistically binds to major histocompatibility (MHC) II complexes, thereby blocking and/or displacing myelin antigens from presenting to T cells [12,13]. GA further exerts its effects by altering the differentiation of T cells—preferentially stimulating T-helper 2 (Th2) over T-helper1 (Th1) cells [14]. Th1 cells are critical for effective immune responses against acute infection, injury, and tissue damage, and are responsible for inducting the innate cellular immune and phagocytic responses. Th1 cells are typically also implicated in the pathogenesis of autoimmune processes [15]. Th1 cells’ function includes the release/stimulus of proinflammatory cytokines including interleukin- 12 (IL-12) (inhibits Th2 cells, increases macrophages), IL-18 (induces IFN-γ, monocytes, macrophages, and dendritic cells), IFN-γ, and TNF-α [16]. T-helper 17 cells (Th17) are also known to induce an inflammatory immune response via proinflammatory cytokines such as IL-17 and INF-γ. A 2020 study examined the potential effects of GA against CD4^+^ Th17 cells and the cytokines they produce. Results from these in vitro experiments show that GA is successful in suppressing and/or decreasing Th17 cells and their associated proinflammatory signaling pathways [17]. Moreover, Th1 and Th17 cell subtypes both exert proinflammatory responses and are involved in tissue injury [13,17].

Conversely, Th2 cells have an anti-inflammatory response. GA-specific Th2 cells can cross the blood–brain barrier (BBB) and release anti-inflammatory and protective cytokines such as IL-4, IL-5, IL-10, TGF-β, and IL-13, all of which can terminate an immune response and mediate tissue repair and regeneration [15]. Interestingly, studies have demonstrated that GA-activated Th2 cells increase the secretion of protective neurotrophic factors including insulin-like growth factor-1 (IGF-1), IGF-2, and brain-derived neurotrophic factor (BDNF) [18,19,20]. Additionally, in RRMS patients, GA is shown to elevate the prevalence and function of T regulatory (T_reg_) cells as well as activation of *FOXP3*, a gene which helps regulate the immune system. T_reg_ cells have an immunosuppressive effect which leads to immune regulation and homeostatic maintenance [16]. Similarly, B regulatory cells (B_reg_) suppress autoimmune pathologies, pathogenic T cells, proinflammatory cytokines and stimulate/produce anti-inflammatory cytokines IL-10, IL-35, and TGF-β. Both T_reg_ and B_reg_ cells’ regulatory effects on the immune response lead to self-tolerance and/or immunological tolerance. GA was additionally found to downregulate granulocyte–macrophage colony-stimulating factor (GM-CSF), which typically functions as a cytokine by stimulating granulocytes and monocytes. A downregulation of GM-CSF was correlated with an elevation in IL-10, Th2 cells, T_reg_ cells, and B_reg_ cells [16].

Recent studies have found that GA has broader immunomodulatory effects on both central and peripheral immune systems [21]. Importantly, in MS patients treated with GA, monocytes were seen to cross the BBB into the brain parenchyma and differentiate into immunoregulatory macrophages [12]. GA is shown to increase and augment the phagocytic activity of monocytes, both in vitro and in vivo [22]. These experiments found an in vitro phenotypic shift from CD14^+^CD16^−^ monocytes to CD14^+^CD16^+^ monocytes, or intermediate monocytes, which have higher phagocytic activity. Specifically, GA’s effects lead to enhanced recruitment of protective monocytes and directly modulated microglia as well as an increase in IL-10 and a decrease in TNF-α [22,23]. Overall, GA promotes and improves phagocytic activity of monocytes and microglia towards myelin debris [13,22,23]. Thus, GA has been shown to impact the phenotype of myeloid cells, including monocytes and microglia, within the periphery and cerebral microenvironment [18,22,24,25].

One cytokine that has been explored more thoroughly in pre-clinical and clinical studies in relation to RRMS and GA is IL-1/IL-1β and IL-1 receptor antagonist (IL-1ra). IL-1ra is a naturally occurring inhibitor of the proinflammatory cytokine, IL-1. Previous studies have hypothesized the possibility of targeting IL-1ra in inflammatory and autoimmune disease therapies [26,27]. Studies found that IFN-β, an alternative therapy for RRMS, was able to modulate the serum levels of IL-1ra which were within normal range in remitting phases, elevated during exacerbations, and elevated after 6 months of IFN-β treatment [27]. More recent studies explored GA’s effect on IL-1β and IL-1ra [10,26]. IL-1β, alongside various cytokines such as IL-19, IL-6, and TNF-α, is known to initiate the innate immunity and is a key mediator of the immune response [10]. In an animal model of RRMS, increased IL-1ra levels were shown to improve disease outcomes. Importantly, in this study, GA was shown to strongly diminish IL-1β expression and enhanced IL-1ra [26].

GA has also been shown to inhibit a very specific receptor, purinergic P2X7 ionotropic receptor (P2X7R), which is found to be increased in inflammatory states, specifically MS. P2X7R is a receptor expressed on monocytes and microglia and is imperative in the activation and proliferation of microglia, potentially leading to destructive, repetitive neuroinflammation and tissue damage. It is also associated with the production of several cytokines responsible for initiating the innate immune response. This clinical study examined GA’s potential effects against this receptor, and it found that GA downregulated P2X7R and its associated inflammatory effects [10].

Although counterintuitive, microglial inflammation is an important negative regulator of the neurogenic microenvironment, as microglia uniquely can both support and interfere with synaptic and neuronal processes [28]. How microglial cells respond to their environment can be influenced by several different factors, not all of which are fully understood. Depending on the environment, GA has been shown to enhance the proinflammatory effects on monocytes in the periphery as well as induce phenotypic shift of brain microglia to both the pro- and anti-inflammatory profiles [18]. For example, GA displayed a direct modulation of microglia cells, leading to phagocytosis [23]. Additionally, there is a bystander expression of anti-inflammatory cytokines such as IL-10 and TGF-β by resident astrocytes and microglia. In fact, there are several central outcomes seen with GA administration beyond phagocytosis of myelin debris. GA is shown to augment remyelination, improve axonal length, increase proliferation of oligodendrocyte progenitor cells, and increase proliferation and differentiation of neuronal progenitor cells [13].

Importantly, GA does not appear to suppress the peripheral immune response as so many Disease Modifying Therapies (DMT) typically do. Instead, this copolymer appears to have an immunogenic effect and enhances the protective peripheral and central immune responses [11]. Studies have demonstrated that a complete suppression of the immune system is not productive for long-term neuronal health [29]. In fact, recent reports have shown that this can later lead to exacerbations of neurodegenerative disease progression in the brain [18]. GA-mediated autoreactive T cells have expressed protective autoimmunity within the brain parenchyma leading to neuroprotection [9]. GA is currently administered subcutaneously at 20 mg daily or 40 mg thrice weekly. This regimen allows for immunomodulation, inflammatory suppression, and peripheral tolerance [13]. Interestingly, this regimen is well-tolerated in RRMS outcomes but not in other disease states in which GA’s role is being explored [13].

### 1.2. Current and Potential Uses of Glatiramer Acetate

As it affects separate aspects of the immune system, GA is a suitable option for targeting several components of MS pathogenesis, and perhaps other neuro-inflammatory conditions [25,29]. Emerging studies are gaining a new understanding of GA’s mechanism of action—one that is not just immunogenic or immunomodulatory but also includes a neuroprotective effect [30]. These effects are believed to be exerted in a multitude of ways including reduction in CNS injury by modifying innate and adaptive immune cell phenotypes. These in turn can lead to prevention of demyelination, inhibition of motor neuron loss, protection against ischemic changes and reduction in scar tissue formation, as well as elevated secretion of neurotrophic factors promoting synaptogenesis and neurogenesis [31,32]. Indeed, GA may aid in resolving both acute and chronic neurodegenerative lesions by enhancing neurogenesis and synaptic plasticity [32,33]. More specifically, studies have demonstrated that GA-activated Th2 cells increase the secretion of insulin-like growth factor-1 (IGF1) and brain-derived neurotrophic factor (BDNF) [18,19,20]. BDNF is critical for neuronal and glial cell differentiation and survival and for axonal protection. It can restrict neuronal damage and promote repair [19,20]. Interestingly, BDNF has been tightly linked with cognitive function and studies show that there are lower levels of BDNF in the brains of MS patients, which is hypothesized to be correlated to MS-related cognitive deficits [34,35]. These new findings are most relevant for the potential of GA to exert neuroprotection and preservation of cognitive function in various neurodegenerative and neuroinflammatory conditions. This is a novel concept that is on the forefront of current research. In this review, we cover findings from numerous pre-clinical and clinical studies utilizing GA under various neurodegenerative conditions.

## 2. GA in Clinical Trials

### 2.1. Role of GA in Preventing Cognitive Decline in Multiple Sclerosis

Since GA is known to have therapeutic effects in MS, other aspects of the disease beyond the inflammatory progression were examined. As previously mentioned, BDNF levels in the brain have been proven to be significantly lower in individuals with MS and have been associated with brain atrophy and cognitive impairment [34]. Indeed, there are a growing number of studies suggesting that GA has protective effects on cognitive functioning. Twelve clinical trials were conducted, all of which utilized several assessments to ascertain the link between GA and neurocognitive protection and improvement, as summarized in Table 1. GA’s effect on both motor function and cognition were analyzed. The expanded disability status score (EDSS) [36], a test that approximates the degree of MS-related motor dysfunctionality via ambulatory status, was frequently used to correlate cognitive findings to disease state.

Table 1 delineates the various outcomes of these twelve clinical studies in MS patients following GA treatment. The results of several assessments showed improvements in physical disability, higher reported quality of life, and reduced levels of fatigue and stress [45,46,47,54]. Additionally, GA showed signs of enhanced information-processing speed and working memory [42,52]. In fact, multiple aspects of memory, including short-term, working, and long-term, were preserved in GA-treated test subjects across three studies [49,50,52].

MS is not only linked to a decline in cognitive processes such as memory, executive functioning, and comprehension but also to psychological issues, primarily depression [55]. Studies have found that depression and its concomitant conditions significantly affect MS patients [55]. Despite this, there are few standardized approaches to diagnose and treat MS-related depression [56]. However, in four out of twelve studies analyzed, GA was found to decrease depression rates in MS and displayed a reduction in comorbidities associated with MS and depression, such as fatigue [45,46,50,52].

Eight of the twelve studies analyzed found GA-driven improvements in multiple cognitive domains including comprehension, evaluation, and analysis of complex situations, and synthesis of appropriate responses [42,45,46,47,49,50,52,54], revealing a possible correlation between GA and cognition. Across these studies, GA administration was linked to mild and/or moderate amelioration of cognitive decline in memory, fatigue, evaluation of new information, processing time, critical thinking, synthesis of novel concepts, decision making, and application of ideas [42,45,46,47,49,50,52,54]. This finding is in line with the proposed neuroprotective effects of GA to reestablish neuroplasticity and reverse degenerative and inflammatory lesions [25].

Despite these findings, it should be argued that the data represent modest improvements in cognitive decline in MS and does not show a definitive link between GA and cognitive preservation or improvement. However, it is important to ascertain the research design and methodology utilized to obtain the data. The studies employed various cognitive assessments. Only two studies utilized standardized assessments which can be applied to the general population, such as the Montreal cognitive assessment (MoCA), the Beck depression inventory (BDI) and Center for Epidemiological Studies Depression scale (CES-D) [57,58,59]. The rest of the studies utilized assessments which are specific to MS patients, including: multiple sclerosis inventory of cognition (MUSIC), multiple sclerosis functional composite (MSFC), modified fatigue impact scale (MFIS), fatigue impact scale (FIS), multiple sclerosis impact scale (MSIS-29), functional assessment of multiple sclerosis (FAMS), Brief International, Cognitive Assessment for Multiple Sclerosis (BICAMS), and multiple sclerosis quality of life (MSQoL)-54 [60,61,62,63,64,65,66,67]. These MS-specific assessments have a skewed perspective and fail to consider multifactorial components of cognitive decline, making it difficult to correlate these findings to generalized outcomes. When taking into consideration the limited scope of these assessments, it is worth contemplating the implications this has on future research of GA’s potential use. Additionally, many of these tests can be “learned”, meaning that once a participant is administered a cognitive assessment, they are able to retain some of the information and can perform better when given the test at a later time to track progression. Thus, participants’ performance might be artificially improved due to learning of the test and not actual improvements from the tested therapy.

**Figure 1:**
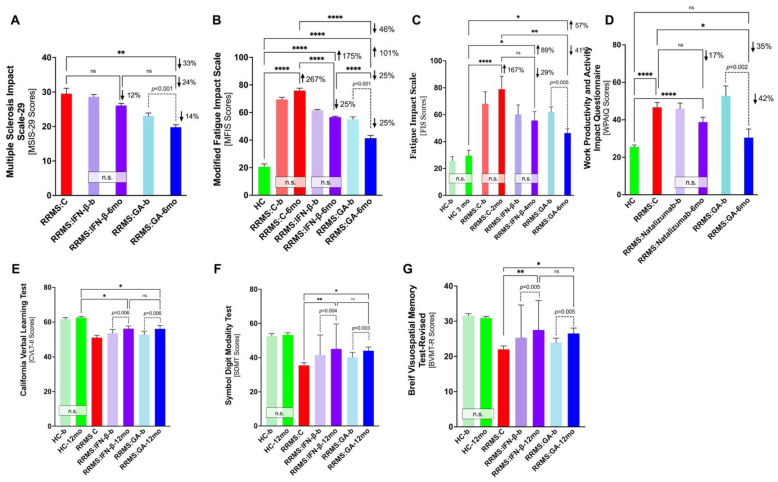
Cognitive and Behavioral studies involving RRMS patients following GA immunization treatment. (**A**) MSIS-29 examines the physical, cognitive, and psychological impacts of multiple sclerosis on participants’ lives. Statistically significant improvement between RRMS controls and GA after 12 months group with a 33% decrease. A statistically significant improvement between GA baseline and 12 months of GA treatment, with a 14% decrease (*p* < 0.001). No significant change in INF-β-treated RRMS cohort. There was a 12% decrease in scores between RRMS controls and INF-β treated as compared to 33% decrease between RRMS controls and RRMS GA-treated cohort. (**B**) MFIS examines fatigue. Statistically significant improvement between RRMS controls and GA after 12 months group with a 46% decrease. A notable improvement between GA baseline and 12 months of GA treatment, with a 25% improvement (*p* < 0.001). No significant change in RRMS controls and INF-β-treated cohorts. (**C**) FIS examines fatigue. Statistically significant improvement between RRMS controls and GA after 12 months group with a 35% decrease. An even more notable statistically improvement between GA baseline and 12 months of GA treatment, with a 45% increase (*p* = 0.002). No significant change in natalizumab treated RRMS cohort. Additionally, no significant difference between healthy controls 6 months of natalizumab treatment. (**D**) WPAIQ examines productivity and disease impact on activity/productivity. Important to note, no significant difference between healthy controls and GA-treated RRMS patients. Statistically significant improvement between RRMS controls and GA after 12 months group with a 35% decrease. An even more notable statistically improvement between GA baseline and 12 months of GA treatment, with a 45% increase (*p* = 0.002). No significant change in IFN-β treated RRMS cohort. Additionally, no significant difference between RRMS controls and 6 months of IFN-β treatment. Graphs (**E**–**G**) represent the 3 tests that make up the BICAMS. (**E**) CVLT-II examines verbal learning and memory. No significant change in healthy controls. A 19% decrease in scores between healthy controls at 12 months and RRMS controls as compared to 10% decrease between healthy controls and RRMS GA-treated cohort. Statistically significant improvement between RRMS controls and GA after 12 months group with a 25% increase. No statistical difference between INF-β-treated cohorts at 12 months and GA-treated cohorts at 12 months. Statistically significant increase/improvement/change between GA baseline and 12 months of GA treatment (*p* = 0.006). (**F**) SDMT, a test of short-term, visual, and working memory. No significant change in healthy controls. A 34% decrease in scores between healthy controls at 12 months and RRMS controls as compared to 18% decrease between healthy controls and GA-treated RRMS cohort. Statistically significant improvement between RRMS controls and GA after 12 months group with a 25% increase. No statistical difference between INF-β-treated cohorts at 12 months and GA-treated cohorts at 12 months. Statistically significant increase/improvement/change between GA baseline and 12 months of GA treatment (*p* = 0.003). (**G**) BVMT-R of visuospatial memory. No significant change in healthy controls. A 29% decrease in scores between healthy controls at 12 months and RRMS controls as compared to 14% decrease between healthy controls and GA-treated RRMS cohort (nearly half the percent change). Statistically significant improvement between RRMS controls and GA after 12 months group with a 21% increase. No statistical difference between INF-β-treated cohorts at 12 months and GA-treated cohorts at 12 months. Statistically significant increase/improvement/change between GA baseline and 12 months of GA treatment (*p* = 0.005). * *p* < 0.05, ** *p* < 0.01, **** *p* < 0.0001. ns: no significance.

Unfortunately, most of the studies are observational and/or utilize a retrospective research design and have not examined real-time effectiveness of GA. For this reason, we performed statistical analyses of cognitive test outcomes amongst several study groups. A careful review was undertaken to identify studies that had utilized the same cognitive assessments and similar study designs of the GA studies. Participants in the studies were age-, sex-, and ethnicity-matched to the participants in the baseline GA study, as well as matched within disease-specific parameters including disease severity (per EDSS), years since disease onset, form of MS (RRMS exclusively), etc. The cohorts examined in these studies were healthy controls, non-GA-treated RRMS controls, and other treatment RRMS. IFN-β was commonly used as the “other treatment” since, like GA, it is utilized as a first-line therapy for RRMS, is also immunomodulatory and is considered an older drug in RRMS [68]. The extensive statistical analyses (one-way ANOVA, paired—between groups with the same participants, i.e., GA-treated—and unpaired post-hoc analysis, etc.) from the comparisons of these articles are displayed in Figure 1A–G. The figures graphically show these variations utilizing mean scores and standard error means to calculate group comparisons.

Meca-Lallana et al. examined changes in GA-treated RRMS patients’ cognition over six months using three separate cognitive assessments: MSIS-29, MFIS, and the Work Productivity Activity Impact Questionnaire (WPAIQ) [47,69]. The MSIS-29 examines the physical, cognitive, and psychological impacts of multiple sclerosis on participants’ lives; Figure 1A displays the statistical analyses between three studies [62,70,71]. The results of cross-cohort comparisons between MFIS scores, a test for physical and cognitive fatigue, are displayed in Figure 1B [66,72,73,74]. FIS scores, an older version of the MFIS, were compared from four studies and the results are displayed in Figure 1C [64,73,75,76,77]. Group comparisons of WPAIQ scores, which represent disease impact on activity/productivity, are displayed in Figure 1D [69,78,79]. Natalizumab, a newer biologic medication often utilized in refractory/severe RRMS, was the alternative treatment in this comparison [80].

Importantly, there were stable patterns amongst the statistical analyses of scores from each of these cognitive tests. One commonality was that there was no significant change in INF-β-treated cohorts’ scores (or natalizumab in the WPAIQ) from baseline to completion of each study. Additionally, IFN-β and natalizumab treatment had no statistical improvement in scores as compared longitudinally to RRMS controls. This implies that alternative treatment for RRMS has no effect on cognition in these studies. When comparing RRMS controls to GA-treated participants longitudinally, GA participants had a statistically significant improvement in cognition, ranging from 33 to 46% improvement. In the WPAIQ, there was no significant difference between healthy controls and GA-treated RRMS patients after 6 months—conveying the potential for GA to improve scores to the level of healthy controls. Finally, the most substantial and remarkable trend amongst these cognitive tests was seen between RRMS patients’ scores at baseline and after 6 months of GA therapy. These same group comparisons had statistically significant improvements in mean scores of the MSIS-29, MFIS, FIS, and WPAIQ (*p* < 0.001, *p* < 0.001, *p* = 0.005, *p* = 0.002; Figure 1A–D).

Cinar et al. examined the changes in BICAMS scores between RRMS patients after twelve months of GA use as compared to healthy controls and INF-β [50] but did not compare to non-GA-treated RRMS controls. Therefore, an additional article was reviewed that studied cognition in GA treatment naïve RRMS participants via the BICAMS test over 12 months [81]. The BICAMS is comprised of three tests that assess different cognitive domains. The California Verbal Learning Tests II (CVLT-II) examines the cognitive domains of verbal learning and memory; results are displayed in Figure 1E [82]. The Symbol Digit Modality Test (SDMT) tests short-term, visual, and working memory; results are displayed in Figure 1F [83]. The Brief Visuospatial Memory Test-Revised (BVMT-R) examines the cognitive domain of visuospatial memory; results are displayed in Figure 1G [84].

Across all three BICAMS tests, several trends emerged. For example, there was a nearly 50% difference in the average score decrease seen between healthy controls and RRMS controls (19–34%) and the decrease seen amongst healthy controls and GA RRMS patients (10–18%), meaning a smaller deviation from healthy controls following GA administration. Additionally, there was a statistically significant increase (21–25%) in mean scores between GA RRMS patients and naïve RRMS controls, with GA-12 months participants scoring 21–25% better on each cognitive test. Conversely, INF-β was shown to have similar trends in its effects on cognition as compared to GA. However, both INF-β and GA displayed improved cognition after 12 months of use. Specifically, there were highly statistically significant improvements in same group comparisons of GA at baseline and GA 12-months seen in each assessment, the CVLT-II, SDMT, and BVMT-R (*p* = 0.006, *p* = 0.003, *p* = 0.005).

Overall, the findings from this meta-analysis display the propensity of GA to improve and/or preserve various cognitive domains when compared to healthy controls, RRMS controls, IFN-β therapy, and/or natalizumab therapy. Thorough statistical testing across multi-cohort studies repeatedly displayed cognitive improvements within GA-treated patients in longitudinal same group comparisons and when compared to other cohorts. To see GA consistently improve cognition, as compared to several cohorts, across multiple studies is promising for ongoing research.

It is important to consider the scope of these cognitive changes associated with GA. A commonly held counterargument to the articles that found mild/moderate improvement in cognition with GA use is that GA has little or no effect on cognition [85]. Two articles compared GA to other established RRMS therapies and were unable to establish a statistically significant difference between the therapies’ effect on RRMS-related cognitive decline [86,87]. One study found that GA’s effect was similar to IFN-β in improving cognition and the improvements were mild [88]. An additional study found there was no measurable decline or improvement in cognition in the patient groups treated with GA, challenging GA’s potential effectiveness in protecting cognition [38]. Yet another study found that cognitive functioning was stable across ten years in GA-treated patients [40]. Findings such as these could be argued multiple ways. Either GA has no effect on cognition and there are no improvements with continued use, or alternatively, GA is protective against cognition deficit and can prevent decline seen in RRMS.

Thus, multiple issues are presented when studying GA’s effect on cognition in RRMS. It is difficult to establish when cognitive decline occurs at disease onset, before disease onset, after disease onset, etc. [89]. Similarly, the natural history as well as pathophysiology of cognitive decline in RRMS needs to be considered and better understood when studying GA’s potential use. Previous and ongoing research examines these relationships with promising findings, such as correlations between MS plaques and cognitive decline [89]. However, more understanding is necessary to explore therapeutic options. Otherwise, it will continue to be difficult to ascertain how to target and track RRMS-related cognitive decline. It is imperative to understand the process and signs of cognitive decline in RRMS patients for accurate analysis of potential therapies’, particularly GA, effects on cognition.

Overall, the understanding of GA’s use in cognition is complex—while some studies show statistically significant improvement, others show none. There are several reasons to consider why these discrepancies exist including the aforementioned cognitive assessments, the understanding of RRMS-related cognitive decline, and several other confounding variables. Although GA’s cognitive benefits are not robust or consistent across all studies, the fact that it was found is still noteworthy for future studies. Each of these studies consistently stated the need for further research into the role of GA in RRMS-related cognitive decline. The unique MOAs of GA, both neuroprotective and anti-inflammatory, are of great interest. With GA’s potential to improve/protect cognition, it is worth exploring alternative applications of GA.

One of the most common first presenting symptoms of MS is ocular in nature: optic neuritis, which can cause significant vision problems. For this reason, studies have begun to examine the potential benefit of utilizing GA to treat ophthalmic pathologies related to MS. Optical coherence tomography (OCT) was typically utilized to assess retinal nerve fiber layer thickness (RNFLT) and total macular volume, two values that are typically found to be lower in MS patients with ocular signs/symptoms [90]. OCT findings revealed that there was an absence and/or reduction in retinal changes or damage after GA administration [38,40]. These studies found that GA had a beneficial, neuroprotective role in retinal axonal degeneration in MS. GA has been shown to improve MS-associated visual pathology, which is in alignment with the other established use of GA in both MS and ADM [51,53].

GA is already a well-established treatment option for RRMS [91]. The new understanding of GA’s mechanism of action describes an immune-driven protective effect in the central microenvironment against damage and degeneration [21]. The effects of GA are already known to improve MS-related inflammatory processes, resulting in amelioration of physical symptoms associated with the disease pathology [29]. However, as research continues this already well-established copolymer, new roles for its use are being discovered [30]. GA has been shown to not only be protective against inflammation, but also shows potential to have ameliorative effects in MS cognitive decline [25]. Even if these findings are mild, moderate, or inconsistent, it is an interesting concept that could give insight in future research endeavors into the use of GA in not only RRMS-related cognitive decline but other neuroinflammatory or degenerative states.

### 2.2. Therapeutic Roles of GA in Ophthalmic Disorders

Beyond RRMS, GA has become a proposed therapy for the treatment of age-related (adult-onset) macular degeneration (AMD) [24]. AMD is a degenerative disease that occurs when drusen, waste products from retinal rods and cones, accumulate over time in the macula causing changes in central and color vision [92]. An animal model found that mice deficient in monocytes and/or macrophages developed hallmarks of AMD while a clinical trial similarly found a reduction in phagocytic activity in AMD patients [24,92]. Therefore, it was hypothesized that the depletion of monocytes and their phagocytic activity was part of the pathophysiological process of AMD. Monocytes and their phagocytic activity were studied in both in vivo and in vitro experiments. GA was found to enhance phagocytosis in classic monocytes (CD14^+^CD16^−^), and non-classic (CD14^dim^CD16^+^) monocytes in intermediate and advanced AMD. Additionally, non-classic and intermediate (CD14^+^CD16^+^) monocytes were significantly correlated with drusen area. The phenotypic heterogeneity of monocytes after GA immunization appeared to provide protection against drusen formation and reduced established total drusen area. Additionally, GA-mediated Th2 cells were shown to reduce retinal microglial cytotoxicity, likely induced by amyloid [24]. Another AMD study found a decrease in macular plaque formation [93]. One study also examined GA’s effect on cognitive decline in AMD and identified a decrease in cognitive impairment, which was attributed to the GA-induced brain neurogenesis and neuronal survival [94].

GA’s potential role in glaucoma was reviewed in an animal model as well as a clinical trial. Glaucoma has several forms and a multitude of suspected mechanisms of disease. However, it is generally understood that glaucoma occurs due to increased intraocular pressure causing retinal and optic nerve damage. A severe form of glaucoma, known as acute primary angle-closure glaucoma (APACG), occurs when there is an abrupt disruption of aqueous humor outflow causing a rapid increase in intraocular pressure, greatly increasing the risk of blindness [95,96]. A study in APACG patients found that GA administration was inversely correlated with disease progression. In this study, visual fields were improved [96]. Similarly, an animal model of glaucoma induced chronically elevated intraocular pressure in rats, which led to retinal ganglion cell death and optic nerve damage. This study found that GA induced neurogenesis, repressed retinal ganglion cell death, and attenuated functional decline in rats [95].

Overall, AMD and glaucoma studies identified that GA led to drusen reduction and amelioration of clinical signs related to disease progression, such as visual disturbances. Table 2 summarizes both animal models and clinical trials examining GA’s effectiveness in AMD and glaucoma. All the studies that were reviewed displayed a positive correlation between GA administration and improvement in disease progression and/or clinical symptoms.

### 2.3. GA Immunization in Amyotrophic Lateral Sclerosis (ALS)

The only other neuropathological state that has moved to clinical human trials with GA is Amyotrophic lateral sclerosis (ALS). ALS is a motor neuron disease in which the specific mechanism of disease is not known but is thought to be due to inflammation and/or degeneration of motor neurons in the brainstem and spinal cord [97,98]. There are various known and suspected etiologies, with genetics being the most studied cause of the disease process [99].

Few completed human studies examining the effects of GA in ALS have been conducted. In these studies, participants with ALS were given 20 mg of GA either bi-weekly or daily [97,98]. Table 3 outlines the immunomodulatory outcomes of these clinical studies in ALS patients following GA treatment. These studies primarily examined GA’s immune cellular response, both centrally and peripherally [97]. GA was linked to a robust humoral response, leading to enhanced cytokine production [98]. Additionally, it was found that there was improved T-cell proliferation and increased levels of Th2 in these patients after GA administration as compared to controls [97]. The enhanced humoral response caused a preferential increase in anti-inflammatory cytokines [97,98]. Th2 proliferation and expansion also led to a similar anti-inflammatory response. Interestingly, one study found that changes in the dosage and frequency of GA, daily versus twice weekly, led to different outcomes [98]. Daily dosage was found to increase Th2 cytokines and IL-4 levels and diminish IL-10 levels while twice weekly regimens were associated with enhanced Th1 cytokines and IL-10 levels and diminished IL-4 levels. In fact, all the clinical trials had varying GA dosage and frequency depending on the disease state being studied. With this information, it is important to consider the potential need to alter the regimen of GA depending on disease type and state. For example, in the successful clinical trial in MS patients, GA immunizations were given either daily or three times weekly, potentially inducing immune tolerance to CNS antigens. In ALS or AMD patients, trials involving less frequent GA immunization regimens had more success [24,46,93]. It is worth citing another clinical study for which a regimen of 40 mg/day did not show any improvement in ALS patients [100]. This study underlines the importance of continued exploration of GA’s potential neuroprotective effects in multiple dosages, regimens, and disease states.

## 3. Preclinical Studies Using GA in Neurodegenerative Disease Models

As the neuroprotective mechanisms of action of GA are better understood, more studies are being developed to identify its potential novel uses. Its unique mechanisms, while not fully understood, prove to be relevant in several other pathological states outside of MS. This is likely because GA has been shown to improve a broad range of immunocytes both centrally and peripherally. Recent animal studies have shown that increased levels of IFN-γ, which are associated with inflammatory autoimmune diseases, impeded neurogenesis (especially oligodendrogenesis). However, GA raised levels of IL-4 centrally, which then reversed the effects of IFN-γ [101]. IL-4, increased by GA, was also found to attenuate TNF-α production—an important aspect of protective immunity [29]. Overall, these findings in rodent models for neurodegenerative diseases concluded that GA enhanced neurogenesis and improved symptoms of several disease states, not just RRMS.

Recent data continue to display overwhelming evidence of GA’s potential to reduce neuroinflammation, degenerative processes, synaptic and cognitive deficits, and psychiatric burden [31,95,102,103,104]. These findings allow for an expanded exploration of proposed GA uses in several other neurological disease states, including neurodegenerative processes such as Alzheimer’s disease (AD), Parkinson’s disease (PD), and Huntington’s disease (HD) as well as cerebral ischemia and psychological disorders. Some studies even show that if GA is given early in disease course or at onset, it may prevent cognitive decline [99,105].The exploration of the novel use of GA in other central pathologies is still in the early stages of pre-clinical trials/animal studies, allowing researchers to investigate changes in neural tissue after GA administration and correlate it to physical and behavioral exam findings. Early findings across multiple studies show promise for GA’s ability to mitigate disease progression and cognitive loss.

### 3.1. Effects of GA Immunization in EAE Murine Models of MS

Alternative uses of GA in MS continue to be explored via animal studies. While it is well-established that GA works to reduce the inflammatory processes of MS, more information is needed on its other potential benefits. The cognitive effects of GA are beginning to be examined in clinical trials more regularly. However, animal studies continue to allow for neural tissue analysis and easier control of variables. Table 4 summarizes cognitive and motor outcomes of animal experiments in MS models following GA immunization.

The induced experimental autoimmune encephalitis (EAE) mouse model, which replicates multiple sclerosis inflammatory progression, is commonly used [106]. EAE studies allow the assessment of long-term effectiveness of GA in MS. Several of these studies found that mice treated with GA had similar, or in some instances better, cognitive scores compared to naïve, healthy controls [102,111].

Improved cognitive testing scores were seen in various tests such as the Longa Score Scale (LSS), Cross-Maze Test (CMT), and Delayed Non-Matching to Sample T-Maze (DNMSTM) [112,113,114], implying that GA treatment conserved or even improved cognitive functions [107,110]. Specifically, these animal-model studies found, via histological examination of brain tissue, that GA alleviated neuroinflammatory and neurodegenerative damage to the frontal cortex and hippocampus [102,109,110,111]. Since the hippocampus and frontal cortex are both important in executive functions and memory, it stands to reason that a reduction in inflammation in these areas would lead to improved cognitive findings.

Of note, the role of GA in downregulation of lymphocytic infiltration and reactive gliosis was positively correlated to the prevention of long-term neurological deficits [95,113]. After GA administration, astrocytes morphologically resembled their pre-inflammatory state, indicating the possibility of GA’s reversal of inflammatory effects and disease progression [102,109,111]. GA was found to greatly reduce neurological impairments, correlating to lessened cognitive decline, as displayed by improvements in motor as well as cognitive testing [107,109]. Additionally, neuroinflammation and neurodegenerative progression was slowed or even halted completely in some studies, as seen via laboratory tests such as flow cytometry and/or immunohistochemistry and electron microscopy [102,107]. These analyses showed a reduction in proinflammatory mediators and reduced signs of astrogliosis, oligodendrogliosis, inflammation, and destruction in the central microenvironment [102,109,110,111].

Due to its immunomodulation effects leading to neuroprotection, GA mitigated the clinical evolution of RRMS and provided disease stability [102,110]. Improvements were visualized in neuronal survival, axonal growth, remyelination, formation of new synapses, and axonal regeneration [95,106]. Furthermore, GA offered protection against memory decline, cognitive deterioration, and alleviated disability in established cases of EAE models [102,110,111]. GA prevented disease development and cognitive decline with a significant reduction in the pre-existing clinical manifestations in RRMS animal models [102,107,111]. Studies found that GA can not only prevent disease progression, but also conserves and/or enhances cognitive capacities.

### 3.2. Effects of GA in Animal Models of ALS

Three studies were conducted with ALS disease mouse models examining the effect of GA immunization. Table 5 reviews cognitive and motor findings of these animal experiments. Several methods were utilized to induce an ALS disease state: via an artificial increase in levels of the defective human *SOD1* gene, via facial nerve axotomy, or via crossbreeding SOD1 transgenic and non-transgenic mice [97,115].

In one study, motor neurons were examined after GA administration. The findings indicated that GA’s neuroprotective effects extended to motor neurons and motor activity. Specifically, both acute and chronic degeneration of motor neurons was prevented and/or improved. Additionally, GA-treated mice’s lifespan was significantly increased as compared to untreated controls [99].

However, in other ALS models, the findings were less promising. One study found that GA-immunized mice displayed improvements in motor function, with animals reaching approximately 10% of their pre-symptomatic motor activity and demonstrated a significant diminution in disease progression [116]. Importantly, although there were improvements in symptomatic aspects of the disease, the administration of GA did not change the outcome—lifespan was not extended [116]. Another study examined the utilization of TV-5010, a synthetic high-molecular weight polymer formulation of the same amino acids of GA. This study analyzed motor functions and muscle strength, with no significant improvements either [117]. Additionally, there were no appreciable improvements in lifespan in this experiment. However, the study utilized several different dosing regimens of TV-5010 with some variations in findings. This study utilized a synthetic polymer that is similar to GA and had different findings than other similar studies, so its results may or may not be applicable here [117]. Again, further research is needed to determine the optimal dosing regimens (quantity and timing) of GA.

### 3.3. Role of GA in Repair, Regeneration, and Cognitive Preservation in AD-Model Mice

Neurodegenerative diseases are being thoroughly examined as potential targets for GA. Alzheimer’s Disease (AD) is a neurodegenerative disease characterized by chronic inflammation which alters amyloid β-protein (Aβ) metabolism, Aβ plaques and neurofibrillary tangles formation, leading to impairment of synaptic plasticity and cognitive function [118]. This may be the mechanism behind AD’s disease progression, presentation, and cognitive decline [119]. Numerous animal studies have been conducted examining the effects of GA on the degenerative processes associated with AD. Current research shows that AD has similarities to MS in the central degenerative and inflammatory processes [120]. Specifically, mitochondrial injury is typically part of the process causing degeneration of various central microstructures such as neurons, axons, and synapses [121]. Additionally, astrogliosis and microglial activation are very similar in MS (specifically RRMS subtypes) and AD [118,119,121]. Considering the similarities in pathophysiology between these diseases, several studies have been exploring the potential for GA to treat AD.

Studies examining AD have found that the resident immune cells of the CNS are not sufficient in clearing Alzheimer’s-related inflammation and Aβ plaques. However, animal models show that GA-enhanced peripheral immune cells can cause central immunomodulation via elevation of protective anti-inflammatory cytokines [20]. Studies are also examining the potential role of the innate immune system response in targeting AD-associated Aβ accumulation and plaques [122]. The findings from these new and interesting studies could be very beneficial to the understanding of natural immune responses’ effects in neurodegeneration and how GA might be able to assist in this via immunomodulation.

Since GA has been found to boost peripheral immune responses, studies have begun to examine GA’s potential use in AD, including the well-established APP_SWE_/PS1_ΔE9_ transgenic (ADtg) murine model of AD [123]. Cerebral recruitment of specific, protective monocytes is found to be induced by GA, specifically to Aβ lesion. The peripherally derived monocytes are highly active, with roles in Aβ degradation, immune regulation via secretion of anti-inflammatory cytokines and downregulation of proinflammatory cytokines, and neurotrophic support/neuroprotection [32]. GA immunomodulation was found to restore pre- and post-synaptic density and induce both synaptogenesis and neurogenesis, resulting in preservation of cognitive functions [124].

Table 6 summarizes cognitive and motor outcomes in animal experiments of AD models following GA administration. These studies found an increase in the Th2-derived regulatory anti-inflammatory cytokines Interleukin-4 (IL-4) and Interleukin-10 (IL-10), primarily around Aβ plaques, and a reduction in proinflammatory mediators (i.e., TNFα, IL-6) [31,104,105,125]. In fact, several studies found that there is an important role for GA-driven immunomodulation, affecting both the central and peripheral immune responses, leading to regulation and repair causing Aβ removal in AD models [31,32,95,124,126,127]. One study found that GA-activated central immune cells, such as microglia, degraded, engulfed, and cleared soluble fibrillar Aβ plaques [125]. Our group found that these GA-activated microglia, macrophages, and bone-marrow-derived monocytes (MΦ^BM^) aided in degradation of Aβ plaques [32,124] and that GA promoted neuroprotective, phagocytic, pro-healing and anti-inflammatory phenotypes in macrophages [95]. The new phenotypes were associated with proliferation and survival of oligodendrocytes, preserved synaptic processes and increased levels of neural progenitor cells, showing signs of enhanced neurogenesis and neuroprotection [95,127].

AD animal models underwent thorough CNS analyses for AD-like pathology such as Aβ plaques. Neural tissue taken from the GA-treated ADtg mice displayed enzymatic degradation of Aβ plaques as well as reduction in and regulation of central inflammation [31,95,126,127,128]. All the observed ADtg had decreased Aβ_42_ levels, likely due to a GA-stimulated increase in macrophage-aided removal of the Aβ plaques [31,104,105,124,125,127,128]. Specifically, GA was shown to reduce Aβ depositions in the cerebral vasculature, retina, and parenchyma [18,104,105] and was linked to amelioration of AD signs, both in the cerebrum, the retina, hippocampus, brain cortex, and other parenchymal areas [105,124,126,129]. Additionally, levels of MMP9 protein, an enzyme known to degrade Aβ, were increased [31,32]. GA-enhanced immune cells reduced Aβ_42_ oligomers and protected the integrity of synapses and neuronal structure [31,124]. Substantial reductions in Aβ plaque burden were detected after GA immunizations [18,104,105,126,128]. Additionally, GA was found to induce neurogenesis, neuroplasticity, synaptoprotection and preservation, regeneration of the cortical microenvironment and eliminate highly toxic Aβ_42_ and Aβ_40_ oligomers [95,104]. GA induced monocyte recruitment and phenotype shift, causing a regulation of local inflammation and leading to a decrease in vascular and parenchymal Aβ plaque burden [18,124,127]. Similar to previous disease states, GA was found to enhance the expression of IFN-γ and the protective neurotrophic factors, BDNF and IGF-1 in AD [129]. Unlike findings from EAE animal models and RRMS clinical trials, weekly administration of GA was found to reduce Foxp3^+^ Treg levels. Moreover, studies found that GA’s immunomodulation efficiently cleared cerebral Aβ, diminishing astrogliosis and detrimental neuroinflammation [31,32,104,105,124,127,129]. Therefore, GA could mitigate AD’s effects since the drug is able to increase protective peripheral immune cells, modulate T-cell response, and aid in protection of the central microenvironment.

Cognitive functioning was also examined, and a significant statistical decrease in cognitive deficits associated with AD was found. The increase in insulin-like growth factor-1 (IGF-1) that was detected in the brains of mice following GA immunization may further explain the enhanced neurogenesis and cognitive function in these mice. Interestingly, there was also evidence of improvements from baseline in cognitive functioning and protection against decline [31,32,96,101,102,123]. Importantly, GA was also associated with an improvement in cognition, demonstrating that GA has the potential to reverse cognitive decline [31,80,81]. Cognitive domains such as memory, learning, spatial memory, discrimination index and special recognition were assessed. This was performed utilizing various behavioral tests, such as the Longa Score Scale (LSS), Morris Water Maze Test, (MWMT), Radical Arm Water Maze (RAWM), and the Barnes Maze Test (BMT) [103,132,133,134]. Several studies found that rodents displayed stable and enhanced cognitive functioning [18,31,104,126,128,129]. GA protected against cognitive decline and preserved neurofunction, largely due to GA’s robust immunomodulatory and neuroprotective effects.

Overall, these studies found that GA attenuated pathological and neurodegenerative processes in AD animal models. GA’s immunomodulation was linked to expansion of Th2-type cells and increased cerebral recruitment of neuroprotective monocyte-derived macrophages. The recruited monocytes contributed to a phenotype shift of the local cellular and inflammatory milieu, including tilting the balance between levels of pro- and anti-inflammatory cytokines and metalloproteinases—all of which contributed to ameliorating AD pathology [31,32,101,105,125,128]. Evidence continues to show that GA abrogates the accumulation of various toxic forms of Aβ in the CNS [31,104,124]. Importantly, GA mitigates cognitive decline and protects against degenerative processes, at least in part by secreting neurotrophic factors such as TGFβ, OPN and IGF-1, affecting neurogenesis and neurocognition processes [18,31,104,105,126,128]. It is important to note that certain studies compared daily versus weekly administration of GA in AD animal models. Although weekly administration was beneficial, daily injections of GA were detrimental leading to moderately worsened cognition and there was no evidence of Aβ plaque clearance [129]. Due to its dual mechanism of action, immunomodulatory effects and neuroprotective benefits, GA could potentially be a very important component of AD care, targeting neurodegeneration and cognitive functioning.

### 3.4. GA Immunization in Animal Models of Parkinson’s Disease (PD)

Parkinson’s disease (PD) is the second most common neurodegenerative disease, following AD. It is characterized by the degeneration of the dopaminergic neurons within the substantia nigra pars compacta and a reduction in dopamine. This leads to movement deficits, particularly causing impaired initiation of movement [135]. With this understanding of the neurodegenerative pathophysiological process of PD, it could potentially benefit from the neuroprotective effects of GA. Thus, animal studies have recently been conducted to examine this possibility.

Table 7 outlines cognitive and motor outcomes in two animal models of PD following GA treatment. The MPTP (1-methyl-1,2,3,6-tetrahydropyridine) neurotoxin model is commonly used to induce a pathological state similar to PD in mice [136]. Like previous studies, one PD model found that GA led to an increase in BDNF, IL-4 and IL-10, implying neuroprotection [137]. GA was found to improve gait and movement behaviors [137]. Enhancements in motor behaviors were visualized via results from laboratory testing methods [138]. Animals displayed a tendency to explore novel areas of mazes, relating to cognitive improvements, and had improved gait [135]. These studies also identified that GA protects the substantia nigra from PD-related neurodegeneration and motor complications [135,137].

### 3.5. GA Immunization in Murine Models of Huntington’s Disease (HD)

Huntington’s Disease (HD) is yet another neurodegenerative disease that could potentially benefit from GA’s neuroprotective effects. HD is associated with a genetic mutation: a trinucleotide repeat expansion, CAG, in the Huntingtin (HTT) gene of humans. This mutation leads to progressive parenchymal tissue damage causing a broad range of central deficits including sensory, motor, and cognitive [139]. Recent studies exploring the specific mechanisms of degeneration in HD have found increased free radicals, increased excitotoxicity, suspected inflammatory processes, and importantly, altered/lower levels of BDNF [140].

Therefore, mouse models of HD were utilized to assess GA’s effect in HD-like pathological states. Male mice on a B6CBA genetic background and female mice on a FVB background were crossbred. The offspring were tested for CAG nucleotide repeats, which were confirmed with PCR and genotyping [141]. Table 8 examines the cognitive and motor outcomes in animal experiments of HD models after GA immunization. Studies examined BDNF expression and its effect on reducing pathogenic astroglial cells. In these models, GA restored BDNF levels and decreased neurodegeneration [139,140,142]. With GA use, lifespan was prolonged and disease progression was delayed [139,140].

GA also was associated with improved cognitive functioning and motor/neurofunction [139,140,142]. Cognition was preserved by GA, as observed in the open field behavioral analysis (OFBA) and the rotarod tests [147,148]. The rodents in the OFBA tests showed less aggression, more purposeful movements, and improved decision-making [139,140]. The drug was also linked to less severe presentation and a later onset of behavioral issues [136]. GA was additionally found to ameliorate hyperactivity often seen in HD [140]. Motor functions and stereotyped behavior or movements were also improved after GA administration [140,142]. Additionally, the neuroplasticity and neurogenesis enhancements garnered by GA use was clear in these studies. Overall, these studies show that GA could play an important role in future studies of HD treatment.

### 3.6. Role of GA in Neuropsychology

In previous studies, GA has been shown to improve not only cognitive domains but also psychiatric conditions and symptoms. Recent reports have implemented animal models of various psychiatric conditions to evaluate the potential for GA to mitigate their symptoms. Table 9 summarizes the outcomes in animal models of neuropsychiatric pathologies following GA immunization.

One article studied genetically induced immunodeficiency in rodents and then administered psychoactive drugs that have a negative effect on mental status and cognition [103]. This combination of genetic and environmental effects was meant to represent a schizophrenia model, as well as other similar psychiatric conditions. This article found that GA reversed the effects of psychoactive agents, despite a weakened immune system. In particular, test subjects were found to have better communicative behavior as well as memory.

In a study examining stress, rodents were exposed to chronic mild stressors (CMS) via brief periods of oxygen deprivation or small shocks [154]. Levels of nitric oxide synthase (NOS) as well as reactive oxygen species (ROS) were then measured. Higher levels of ROS, which are free radicals, cause damage via oxidative stress [162]. Conversely, NOS is an antioxidant that is associated with protection from destructive processes such as infection, inflammation, and cell death. Therefore, when evaluating the effects of ROS and NOS, ROS will cause neurodegeneration and cognitive deficits [163], whereas NOS is associated with neuroprotection and found in higher levels of anti-inflammatory states [140]. Here, GA’s effects on the brain were examined in relation to stress [154]. Treatment with GA resulted in an increase in NOS and a decrease in ROS, leading to lower rates of negative outcomes and complications from stress and ROS.

One study implemented the use of lipopolysaccharides to cause short-term memory impairments in mice and found that GA injection improved axonal growth and remyelination [157], which correlated with memory improvement and shorter latency times in task completion.

In the model of radiation injury, rodents had significant short-term and spatial memory deficits. However, in treatment groups, GA was linked with restoration of hippocampal neurogenesis [161]. Due to this GA-mediated improvement in neurogenesis and neuroplasticity, multiple aspects of memory including short-term, long-term, and spatial were improved. Additionally, GA was linked to reversal of behavior impairment associated with the radiation.

The neuropsychological models found a positive correlation between GA use and improved memory, communicative behavior, psychosocial interactions, and stress response [98,154,157,161]. These promising results warrant further exploration into potential neuropsychiatric applications of GA. Disease states that GA is known to ameliorate, such as RRMS, often have neuropsychiatric components. Therefore, this drug could potentially serve dual purposes for many disease states: both the psychiatric burdens and inflammatory complications could be targeted, thereby lowering multiple aspects of the morbidity of these diseases.

### 3.7. Role of GA in Central Ischemia and Vascular Dementia

Ischemia within the central nervous system (CNS), particularly within the brain itself, can be caused by a multitude of etiologies. Most commonly ischemic brain injury is due to a thromboembolic stroke and/or cerebral hemorrhaging. Central ischemia can cause significant neuropathological changes. The functional deficits of sustained CNS ischemia are dependent on the area the ischemia is located in. However, clinical signs/deficits are typically motor, sensory, verbal, and cognitive in nature [164]. Cerebral ischemia specifically due to stroke has been shown to increase neural inflammation. This is thought to be due to the breakdown in BBB integrity, leading to an influx of immune cells into the brain. Additionally, a local immune response is triggered by endogenous tPA endothelial release, activating astroglia and microglia [165]. Therefore, several studies have begun to examine what effects anti-inflammatory therapy has on cerebral ischemia complications. Table 10 summarizes cognitive and neurofunctional outcomes in animal models of cerebral ischemia following GA immunization.

In these animal models, cerebral ischemia was induced via several techniques, one of which was permanent middle cerebral artery occlusion (pMCAo) in mice [170], whereby cognitive decline was induced, and inflammation was exacerbated in the brain. This allowed for an analysis of the ischemic pathological state and the potential improvements via GA treatment. Studies specifically examined memory and sensorimotor functioning before and after GA treatment following a cerebral ischemic injury. As seen in several other studies, GA displayed an immunomodulatory effect, with increase in anti-inflammatory mediators [31,164,169]. In nearly all CNS ischemia (CNSi) animal models, the immunomodulatory and neuroprotective effects of GA were linked to an enhancement in early neurogenesis, improved neuroplasticity, and strong neuroprotection. With these improvements, GA was found to prevent long-term memory loss and reduce cognitive deficits.

Vascular dementia was induced via permanent cerebral artery occlusions, similarly to cerebral ischemia [175]. GA was once more found to increase the expression of BDNF and modulate the hippocampal balance of Th1/Th2 cells and associated cytokines [174]. These effects positively correlated to a reduction in cognitive deficits.

In each CNSi study, there was a significant reduction in post-ischemic infarct volume [164,167,169,174]. Novel effects of GA, demonstrated in the reduction in the neurovascular damage to cortical regions, can be related to the immunomodulatory activity of GA. Treatment with this copolymer prevented neurodegeneration associated with ischemic injury and inflammation [164,167,169]. Additionally, GA was associated with an accelerated recovery of sensorimotor functions [164,174]. A significant improvement in neurological functions was identified in GA-treated subjects as compared to controls [167,168,174]. Collectively, these models of vascular dementia and cerebral ischemia demonstrated the benefit of using GA in the early phase following a stroke with signs of improvement in inflammation, memory loss and sensorimotor deficits [164,167,168,169,174].

**Figure 2:**
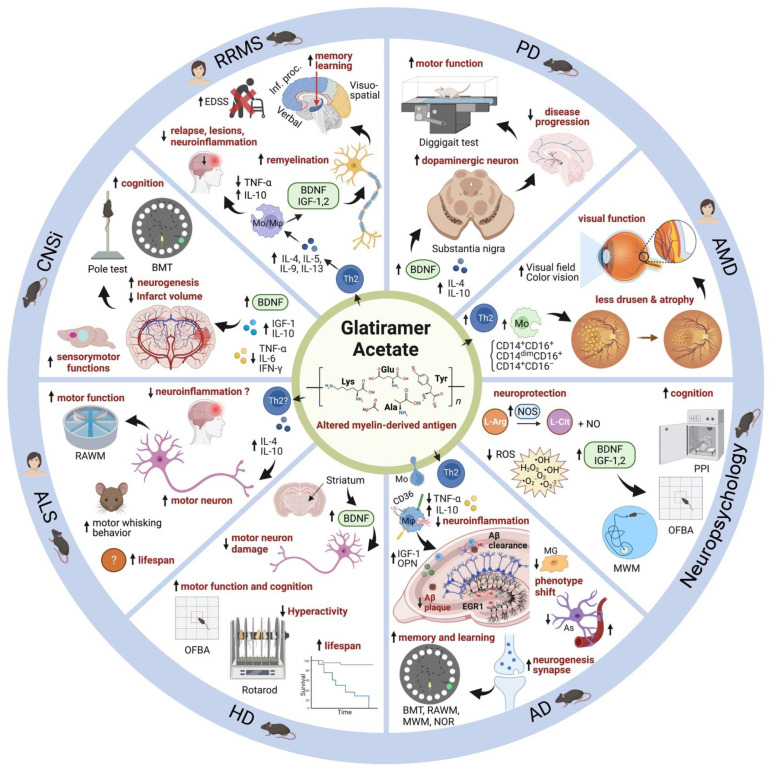
Mechanism of action and therapeutic effects of GA neuroimmunomodulation across various neurologic disorders. The synthetic immunoactive copolymer glatiramer acetate (GA; formula C_25_H_45_N_5_O_13_), branded Copaxone (also known as Copolymer-1 or Cop-1), is comprised of four amino acids, Lysine, Arginine, Glutamic acid and Tyrosine, in random order, resembling myelin basic protein (MBP). In the CNS under injury or inflammatory conditions, MBP level is increased, and GA is considered as its weak agonist. GA causes expansion of specific populations of helper T type 2 (Th2) cells that secrete anti-inflammatory cytokines and recruitment of monocytes-derived macrophages into the diseased brain, spinal cord, and retina. PD: data is based on pre-clinical studies. GA immunization (200 μg/s.c. or 3.5 mg/kg/s.c. daily for four weeks) increased BDNF, IL-4 and IL-10 levels and protected the substantia nigra from dopaminergic neuron degeneration thus limiting disease progression and improving motor functions. AMD: data is based on clinical studies, where GA immunization (20 mg/s.c.) was given once a week for 12–16 weeks. GA was found to enhance the phagocytic ability of classic (CD14^+^CD16^−^) and non-classic (CD14^dim^CD16^+^) monocytes. GA immunization induced a phenotypic heterogeneity of monocytes which seemed to provide a protection against drusen formation. Additionally, GA-mediated Th2 cells were shown to reduce retinal microglial cytotoxicity. Overall, GA reduced retinal atrophy and improved visual functions. Neuropsychology disease: pre-clinical data showed that GA treatment (100–250 μg/s.c./daily or weekly for 1–2 weeks) increased neuroprotection and improved cognition (as demonstrated with various behavioral tests) with elevating levels of NOS and neurotrophic factors (BDNF, IGF-1 and IGF-2) along with decreased levels of ROS. AD: data is based on pre-clinical and in vitro studies where GA immunization (100 μg/s.c./weekly for 4–12 weeks) increased infiltration of CD115^+^LyC6^hi^CD45^hi^-OPN^+^ monocytes to the CNS as well as Th2 population. Infiltrating monocytes-derived macrophages (CD68^+^) and their scavenger receptors (CD36, SCARA1, CD163) contributed to enhancing clearance of Aβ plaques and other Aβ assemblies from the parenchyma and blood vessels. Neuroinflammation in the form of reduced GFAP^+^ astrogliosis and Iba1^+^ microgliosis was reduced along with decreased levels of TNF-α and increase in IL-10 levels. Secretion of neurotrophic factors such as IGF-1, OPN, and increased expression of transcription factor EGR1 enhanced hippocampal synapses and neurogenesis. As a result, a phenotype shift from pro- to anti-inflammatory microglia is observed. Overall, GA reduced cerebral inflammation and improved Aβ clearance, preserved synapses and cognition. Interestingly, GA given daily revealed to be detrimental. HD: pre-clinical data of GA treatment (100–750 μg/s.c./daily, weekly, thrice weekly, or five times weekly for 4–12 weeks) showed elevated BDNF levels in striatal cells, decreased motor neuron damage and hyperactivity and improved motor function and cognition thus overall increasing lifespan. ALS: pre-clinical (100 μg/s.c./daily, weekly, bi-weekly, or monthly for 1–4 weeks) and clinical (5–20 mg/s.c./daily or twice monthly for six months) studies demonstrated immunomodulation by GA leading to Th2 proliferation along with increased levels of IL-4 and IL-10, which may reduce neuroinflammation, preserve motor neurons, and improve motor function, thus possibly prolonging lifespan. CNSi: pre-clinical data (100–200 μg/s.c./weekly or thrice weekly for 1–4 weeks) showed that GA treatment ameliorated neuro-deficit, improved cognition and neurogenesis associated with increased level of BDNF, and anti-inflammatory cytokines (IGF-1, IL-10), decreased pro-inflammatory cytokines (TNF-α, IL-6, IFN-γ). GA was also associated with recovery of sensorimotor functions and reduction in post-ischemic infarct volume. RRMS: pre-clinical (200–250 μg/s.c./daily for 1–3 weeks) and clinical (20–40 mg/sc/daily or thrice weekly for six months to ten years) data demonstrated that GA increased levels of anti-inflammatory cytokines (IL-4, IL-5, IL-9, and IL-13) derived from Th2 cells in the CNS. Increased infiltrating-monocytes-derived macrophages decreased TNF-α and increased IL-10, leading to reduction in neuroinflammation, relapse, and lesions. Elevated levels of neurotrophic factors such as BDNF and IGF-1 and 2 were associated with improved cognitive domains such as information processing, verbal, and visuospatial learning and memory. More importantly, GA prevented the formation of anti-myelin antibodies and thus reduced demyelination and promoted remyelination, axonal growth, regeneration, and improved quality of life such as reducing EDSS, fatigue, and depression. Data are derived from pre-clinical (mouse icon) and/or clinical (human head icon) studies. Aβ: amyloid-β; AD: Alzheimer’s disease; Ala: Alanine; ALS: Amyotrophic lateral sclerosis; AMD: adult-onset macular degeneration; As: Astrocyte; BDNF: brain-derived neurotrophic factor; BMT: Barnes maze test; CD: cluster of differentiation; CNSi: central nervous system ischemia; EDSS: expanded disability status score; EGR1: Early Growth Response Protein 1; HD: Huntington’s disease; IGF: insulin-like growth factor; Glu: Glutamic Acid; Inf. Proc.: information processing; IL: Interleukin; INF: Interferon; L-Arg: L-Arginine; L-Cit: L-Citrulline; Lys: Lysine; Mφ: macrophage; MG: microglia; Mo: monocyte; MWM: Morris water maze; NO: nitric oxide; NOR: novel object recognition; NOS: nitric oxide synthase; OFBA: open field behavioral assessment; OPN: Osteopontin; PD: Parkinson’s disease; PPI: pre-pulse impulse; RAWM: radial arm water maze; ROS: reactive oxygen species; RRMS: relapse-remitting multiple sclerosis; s.c.: subcutaneous; TGF: transforming growth factor; Th: T helper cell; TNF: tumor necrosis factor; Tyr: Tyrosine. Figure was created with Biorender.com (accessed on 16 June 2021).

In summary, Figure 2 describes the current knowledge regarding the molecular mechanisms of GA in eight different neurological diseases outlined in this review, including evidence of therapeutic effects and functional benefits.

## 4. Conclusions and Future Directions

GA has been a first-line treatment to target the uncontrolled, detrimental inflammatory processes found in relapse-remitting forms of MS. However, recent studies have found that GA has more therapeutic benefits than previously thought. With emerging evidence that GA immunization induces cerebral BDNF and IGF-1 expression and neuroprotective effects in the CNS (Figure 2), it is imperative to continue to study its implications in various pathologies. The neuroprotection of GA has been found to assist in preservation of synapses and cognitive function and to be prophylactic against cognitive decline. While these effects are useful in the treatment of MS and its related neurocognitive complications, it is also feasible that GA has additional benefits in other disease states. AMD is an already established condition that GA targets; however, visual pathologies in MS have the potential to be targeted by GA as well. Neuroplasticity restoration and cellular repair is another, well-studied role of GA that has proven to be beneficial in treating degenerative and inflammatory lesions in several pathological states outside of MS, including Alzheimer’s, Parkinson’s, and Huntington’s disease. Additionally, the cognitive findings in these neurodegenerative diseases as well as in neuropsychiatric conditions and cerebral ischemia have the potential to be ameliorated by GA’s neuroprotective effects. A key finding among some studies is that GA may reverse inflammatory damage and improve cognitive function, resulting in improved functional status from baseline after GA administration. However, further study of the GA regimen dosing and frequency for each of these unique diseases and pathologies is needed. For example, in acute presentations a one-time administration or short course of GA may be sufficient but for chronic diseases, more intervention could be necessary.

As the inflammatory and immune cells are key players in all of these diseases and the effects of GA, future studies could potentially evaluate the ongoing role of other, less studied cells. For example, macrophage migration inhibitory factor (MIF) and its homolog D-dopachrome tautomerase (D-DDT) are inflammatory factors with a common receptor, CD74. They are thought to be implicated in the pathogenesis of immunoinflammatory diseases and disease worsening. This is thought to be due to their multi-functional, pleiotropic effects leading to several pro-inflammatory states [176,177,178]. Recent studies have examined the possible role of these cytokines in MS. In an animal model, higher level of MIF and D-DT were correlated to increased EAE disease severity. Conversely, animals that lacked MIF and D-DT had a less severe progression of EAE [176]. Another study examined these cytokines in MS patients with clinically isolated syndrome (CIS). It was found that MIF and D-DT were overexpressed in CD4^+^ T cells of MS patients [177]. Similarly, IL-37 is hypothesized to help determine onset and progression of MS [179]. The findings from such studies display the possibility of targeting MIF and D-DT for pharmacological purposes and even diagnostic markers of disease progression [176,177,178]. Overall, it is imperative that the effects of GA continue to be examined and tested to better understand its myriad of neuroprotective benefits and the potential treatments it could offer.

## 5. Review Methods

To perform this meta-analysis, a thorough and careful literature review was conducted. Articles and studies from peer-reviewed journals were assessed that examined potential novel effects of GA with a focus on cognition. Due to the burgeoning nature of this research concept, both clinical trials and animal studies were considered for the review. The online database and search engine, PubMed, was utilized to search for studies that were in line with this review’s goals. Key words and phrases selected for the PubMed search included: glatiramer acetate and cognitive function, GA cognition, Copaxone cognitive function, GA alternative effects, GA neurodegeneration, GA neuroinflammation cognition, Coplymer-1 cognition, Cop-1 cognition, GA Alzheimer’s disease, GA movement disorders, GA psychology, et cetera. There were approximately 1000 articles produced on average from the search utilizing these words and/or phrases. However, not every article fit the criteria of this literature review. To quickly evaluate the relevance of these articles, the abstract was reviewed. If the abstract expressed a focus on GA and cognition within neurological pathologies, the study was further analyzed. However, if it was found that the article was itself a review paper, meta-analysis, or any other non-experimental paper, it was not accessed for review. Additional exclusion criteria included studies published before 2000 (with the exception of MS clinical trials), studies with several confounding variables (e.g., multiple drugs studied in various patient cohorts), and studies not available in English (the primary language of the reviewer). Out of the approximately 1000 articles populated from the first search, another 100 on average had abstracts which coincided with the topic of interest. Once reviewed more carefully, approximately 25 articles further met the search criteria. Articles that were excluded did not have goals or outcomes that were in line with the purpose of this review. For example, if a study was examining the effects of GA in Alzheimer’s dementia but did not focus on cognition, it was not utilized. In general, articles were excluded that did not focus on cognitive effects of GA in specific neurological disease states. Several articles commented on cognition and GA but only those that specifically studied the relationship between GA and cognition were included in this review. A final exclusion criterion was implemented to evaluate the studies: improved or maintained cognitive performance. With this criterion in mind, an average of 10–15 articles were extrapolated from the search and utilized in this review.

The main information obtained from these articles was constructed into a table to allow for a quick overview of the findings. While assessing these articles, the main points considered for inclusion were as follows: GA’s neuroprotective effect, novel uses for GA, research design and methodology, scoring mechanisms/research tools used (e.g., MFIS, Modified Fatigue Impact Scale), findings, analysis of the suspected mechanism of action for GA and emphasis on neurological and psychological pathologies. The results from each article were analyzed carefully for validity, reproducibility, accuracy, and relevance to the current research question. After reviewing the relevant articles, the information obtained was assessed with the overarching theme of novel uses for GA driving this analysis. Once the information was organized appropriately, a deeper evaluation was completed of the findings. The general findings were reported on and divided into categories based on the potential new target for GA use.

A further analysis was made, specifically within the clinical trials examining cognitive effects of GA in multiple sclerosis. Articles were obtained for the purpose of the review with the above-mentioned methods. However, articles were then further selected with more stringent criteria. If they did not present detailed results and statistical analyses, they were not included. For example, if an article did not include specific results such as mean scores from cognitive testing or p-values from comparison analyses, it was not eligible. Additional articles were then selected that had similar study designs testing cohorts with the same cognitive assessments. For example, articles included in this review almost exclusively examined RRMS patients treated with GA and the cognitive effects were studied via various established assessments. Unfortunately, several of these articles did not have control groups that were well-established or any comparisons to other patient cohorts. Therefore, articles were found to perform a comparison analysis of various groups. Articles were found that included cohorts which were matched by age, sex, education status, disease status (if applicable), disease type (if applicable), and disease duration (if applicable). Cohorts were examined with the following group comparisons in mind: healthy controls, RRMS controls (no treatment), or alternative treatment (typically IFN-γ since this is relatively comparable to GA). Once these articles were identified, a rigorous statistical analysis and comparison was carried out. The digital program, GraphPad Prism, was utilized to run these analyses and obtain values for the group comparisons. Comparisons were made amongst groups within specific cognitive assessments. For example, groups were matched and compared within cohort results for MFIS and then analyses were made for that specific test. A one-way ANOVA was utilized with a post-test Tukey to obtain comparative values and determine the statistical significance of the group variations within individual cognitive assessments. Then, Prism was further utilized to graphically display the statistical findings including standard error means and p-values between the groups. The graphs represented a visual display of the statistical significance of these important findings.

## 6. Side Effects and Safety

Since GA is a relatively old drug that is already widely used, the side-effect profile is well understood and tolerated. Additionally, GA is relatively safe with few, if any, significant, strong risks or contraindications. The majority of side effects found in GA are associated with injection-site reactions (pain, erythema, soreness, swelling, and hard indurations) [180]. Additional side effects include nausea, vomiting, chills, arthralgias, myalgias, neck pain, back pain, dyspnea, chest pain, headache, diplopia, polyuria, weakness, rhinorrhea, fever, sore throat, and tremors [181]. The only reported contraindication is a known hypersensitivity to mannitol or GA itself [180,181]. Overall, GA is a well-tolerated and safe drug with few associated risks.

## Figures and Tables

**Table 1:** Clinical Trials Examining Cognitive Outcomes of Glatiramer Acetate Immunization in Multiple Sclerosis Patients.

Disease State	Research Design and Methodology	Findings	Ref.
**MS**	248 MS patients, EDSS < 5 GA (n = 125) Placebo (n = 126) Longitudinal: years	GA—b vs. GA—2 years: stable or improved EDSS scores Placebo—b vs. placebo—2 years: large variations in EDSS scores Neuropsychological tests (PASAT [37], spatial recall, word list generation, etc.) showed no improvements in GA-treated participants Lack of measurable cognitive decline	Weinstein, A. et al., 1999 [38]
**MS**	251 RRMS patients, EDSS < 5 GA (n = 79) Placebo (n = 74) Longitudinal: 10 years	BRBNT [39] GA—b vs. GA—10 years: <0.5 SD, statistically insignificant BRBNT placebo—b vs. placebo—10 years: decline more than 0.5 SD seen Stable cognitive performance	Schwid, R. et al., 2007 [40]
**MS**	30 RRMS patients Gd^+^ GA (n = 18) Gd^−^: GA (n = 12) Longitudinal: 3 months	PASAT [mean ± SD]: Gd^+^ [42.16 ± 1.33] vs. Gd^−^ [48.92 ± 1.51] (*p* < 0.05) iTBS induced LTP-like response [41] [mean ± SD]: Gd^+^ [1.38 ± 1.73] vs. Gd^−^ [1.51 ± 2.59] (*p* < 0.05) Improved cognition (PASAT, LTP) correlated to reduced Gd^+^ lesions	Mori, F. et al., 2012 [42]
**MS**	67 RRMS patients GA (n = 67) Observational study Longitudinal: 24 months	FIS [mean ± SD]: GA—b [61.96 ± 31.04] vs. GA—24 months [45.94 ± 27.54] 26% decrease (*p* < 0.001) MSQoL-54 [mean ± SD]: GA—b [19.3 ± 3.69] vs. GA—24 months [21.8 ± 4.43] Decreased fatigue and improved QoL; remained decreased/improved	Jongen, P. et al., 2014 [43]
**MS**	37 MS patients, no prior use of DMT [44] GA (n = 23) Placebo (n = 14) Longitudinal: 12 months	EDSS GA—b vs. GA—12 months: decreased scores (*p* = 0.003) Placebo—b vs. placebo—12 months: increased scores (*p* = 0.008) MSFC GA—b vs. GA—12 months: increased scores (*p* = 0.0001) Placebo—b vs. placebo–12 months: lowered scores (*p* = 0.0001) MoCA GA–b vs. GA–12 months: no significant change (*p* < 0.083) Placebo–b vs. placebo–12 months: significantly lower scores (*p* < 0.025) Improved cognition in MSFC and MFIS; maintained cognition in MoCA scores.	Vacaras, V. et al., 2014 [45]
**MS**	428 RRMS patients, EDSS < 5.5, GA Observational study Control group: meta-analysis of general MS population statistics	Depression prevalence: GA 13.4% vs. gen. MS population 36–54% Lower depression (BDI scores) correlated w/higher MSQoL-54 EDSS: lower median score Reduced disease activity, antidepressant effect, and improved QoL.	Fricksa-Nagy, Z. et al., 2016 [46]
**MS**	RMMS patients, previously on INF-β w/MFIS > 38 Observational study GA (n = 54) Longitudinal: 6 months	MFIS GA–b vs. GA–6 months [mean ± SD]: Physical: [27.6 ± 4.8] vs. [20.0 ± 7.3] (*p* < 0.001) Cognition: 21.9 ± 8.4 vs. 17.5 ± 7.2 (*p* < 0.001) Psychosocial.: 5.6 ± 1.8 vs. 3.9 ± 1.9 (*p* < 0.001) WPAIQ GA–b vs. GA–6 months [mean ± SD]: Activity impairment: [63.1 ± 23.1] vs. [42.0 ± 23.3] (*p* < 0.001) MSIS-29 GA—b vs. GA—6 months [mean ± SD]: Physical: 51.2 ± 13.3 vs. 44.8 ± 12.0 (*p* < 0.001) Psychological: 23.1 ± 6.0 vs. 19.8 ± 5.3 (*p* < 0.001) Amelioration in fatigue. Improved QoL, cognition and work/daily activities.	Meca-Lallana, J. et al., 2016 [47]
**MS**	754 MS patients Observational study Previous DMT treatment, started GA (n = 481) Treatment naïve, started GA (n = 273) Longitudinal: 2 years	GA—b vs. GA—2 years Relapse rate: 87% vs. 49% (*p* < 0.001) PASAT [mean]: [41.63] vs. [45.76] (*p* < 0.001) MSFC: 64.2% improved, 35.8% deteriorated FSMC [48]: 43.6% improved, 51.3% deteriorated, 5.1% unchanged FAMS: 51% improved, 47.1% deteriorated, 1.9% unchanged MUSIC: 56.5% improved, 26.7% deteriorated, 16.8% unchanged CES-D: reduced depressive symptoms (*p* < 0.001) Mitigated disease progression; improved cognition; reduced depression.	Ziemssen, T. et al., 2016 [49]
**MS**	MS patients, GA-treated (n = 161) Naïve healthy controls (n = 102) Longitudinal: 12 months	BICAMS GA—b vs. GA—12 months [mean ± SD]: SDMT: [40.8 ± 20.5] vs. [44 ± 16.4] (*p* = 0.003) CVLT-II: [52.7 ± 14.8] vs. [56.1 ± 14.3] (*p* = 0.006) BVMT-R: [23.9 ± 10.4] vs. [26.5 ± 11.6] (*p* = 0.005) Improved cognition and slowed onset of cognitive impairments.	Cinar, B. et al., 2017 [50]
**MS**	19 RMMS patients, GA-treated Observational study Longitudinal: 2 years	OCT: reduction in signs of retinal inflammation w/GA Reduced neurodegenerative processes in the retina	Sazonov, D. et al., 2018 [51]
**MS**	33 MS patients, GA-treated Observational study Longitudinal: 4 years	PASAT: improved information processing/speed and working memory	Shorobura, M., 2018 [52]
**MS**	RRMS patients, GA-treated (n = 60) Naïve healthy controls (n = 40) Longitudinal: 2 years	EDSS [mean ± SD]: [2.0 ± 1.0–3.5] vs. [2.5 ± 1.5–3.5] Relapses [mean ± SD]: [0.18 ± 0.46] vs. [0.36 ± 0.58] OCT imaging, RNFLT [mean]: [86.5] vs. [92.3] (*p* = 0.046) OCT imaging, TMV [mean]: [0.67] vs. [0.93] Reduced damage in RNFLT, similar findings to healthy controls	Zivadinov, R. et al., 2018 [53]

Participants in the treatment groups of these studies were given 20 mg/s.c./qd of GA (subcutaneous, daily); MS: multiple sclerosis; RMMS: relapsing remitting multiple sclerosis; GA: glatiramer acetate; EDSS: expanded disability status scale; BRBNT: brief repeatable battery of neuropsychological tests; RR: relapse rates; Gd^+^: gadolinium positive; Gd^−^: gadolinium negative; HRQoL: (health-related quality of life); iTBS: intermittent theta burst stimulation; BDNF: brain-derived neurotrophic factor; PASAT: paced auditory serial addition test; MRI: magnetic resonance imaging; LTP: long-term plasticity; DMT: disease-modifying therapy; MSFC: multiple sclerosis functional composite; MFIS: modified fatigue impact scale; MoCA: Montreal cognitive assessment; MSQoL-54: multiple sclerosis quality of life-54; BDI: Beck depression inventory; INF-β: Interferon-β; WPAIQ: work productivity and activity impairment questionnaire; MSIS-29: multiple sclerosis impact scale-29; MSIC: multiple sclerosis inventory cognitive scale; CES-D: Center for Epidemiological Status-Depression; FAMS: functional assessment of multiple sclerosis; FSMC: fatigue scale for motor and cognition; MUSIC: multiple sclerosis inventory cognition; BICAMS: brief international cognitive assessment for multiple sclerosis; w/o: without; SD-OCT: spectral domain—optical coherence tomography; RNFLT: retinal nerve fiber layer thickness; TMV: total macular volume; SD: standard deviation.

**Table 2:** Clinical Trials and an Animal Model Examining Alternative Outcome of Glatiramer Acetate Treatment in Ophthalmological Patients.

Disease State	Research Design and Methodology	Findings	Ref.
**AMD**	17 AMD patients GA-treated (n = 4) Placebo (n = 4) Longitudinal: 12 weeks	TDA, GA—b vs. GA—12 weeks (mean): (48,130) vs. (16,205), improved TDA, placebo—b vs. placebo—12 weeks (mean): (32,294) vs. (32,781), no significant change Reduced TDA	Landa, G. et al., 2008 [93]
**Glaucoma (animal model)**	8-week-old m Lewis rats elevated IOP (glaucoma model) GA vs. PBS and naïve control (n = 6 per group)	Increased *Egr*, potential GA-induced repair mechanism Five altered genes in elevated IOP rats (*Cspg2*, *Fbn1*, *Enpp2*, *Ncam1* and *Stat1*) were restored to homeostatic levels Induced neurogenesis and cell migration/communication Repressed cell death, scar tissue formation, immune response, and protein degradation Prevention of RGC death and attenuation of functional decline	Bakalash et al., 2011 [95]
**AMD**	14 AMD patients GA-treated (n = 7) Placebo (n = 7) Longitudinal: 12 weeks	Drusen shrinkage rate, GA—12 weeks vs. placebo—12 weeks: 27.8% vs. 6.8% (*p* = 0.008) Drusen disappearance, GA—12 weeks vs. placebo—12 weeks: 19.2% vs. 6.5% (*p* = 0.13) Reduced drusen	Landa et al., 2011 [94]
**Glaucoma**	38 glaucoma patients GA-treated (n = 19) Placebo (n = 19) Longitudinal: 16 weeks	Visual field mean deviation: GA, improved (*p* = 0.01) vs. placebo, worsened (*p* = 0.004) Less disease progression and improved visual fields	Fan et al., 2019 [96]
**AMD**	104 AMD patients iAMD GA-treated (n = 72) iAMD GA-treated (n = 32) Healthy controls (n = 74) Longitudinal: 15 weeks	GA—12 weeks vs. healthy controls—15 weeks: enhanced phagocytosis of non-classical monocytes (*p* < 0.0001) and classical monocytes (*p* = 0.0002) GA—12 weeks vs. healthy controls—15 weeks: reduced drusen and retinal atrophy, iAMD (*p* = 0.02); late AMD (*p* = 0.078) Improved monocyte activity/phagocytosis—correlated to drusen levels and retinal tissue integrity	Gu, B. et al., 2021 [24]

Participants in the treatment group of these studies were given GA 20 mg/s.c./qw (weekly); AMD: age-related macular degeneration; TDA: total drusen area; iAMD: intermediate adult-onset macular degeneration; lAMD: late adult-onset macular degeneration; IOP: intraocular pressure; RGC: retinal ganglion cell.

**Table 3:** Clinical Trials Examining Alternative Glatiramer Acetate Uses in Amyotrophic Lateral Sclerosis Patients.

Disease State	Research Design and Methodology	Findings	Ref.
**ALS**	30 ALS patients GA-treated, qd (n = 20) GA-treated, q2w (n = 20) Placebo (n = 10)	GA: protective T-cell proliferation increased compared to placebo (*p* = 0.02) Destructive immune cell lines diminished Immunomodulatory effects enhanced neuroprotection	Gordon, P. et al., 2006 [97]
**ALS**	31 ALS patients GA, qd (n = 10) GA, q2w (n = 10) Treatment naïve (n = 11) Longitudinal: 6 months	Inverse correlations in IgG3 and IL-4 and IL-10 levels qd GA: enhanced Th2 cytokine levels q2w GA: enhanced Th1 cytokine levels qd GA: diminished IL-10 levels q2w GA: diminished IL-4 levels; increased IL-10 levels Improved protective immune response. Findings varied based on dosage/frequency of GA administration.	Mosley, R. et al., 2007 [98]

Participants in the treatment groups of these studies received 20 mg/s.c.; Monthly plasma samples obtained in ALS models; ELISA and flow cytometry utilized to assess for immune responses; ALS: amyotrophic lateral sclerosis; qd: daily; q2w: biweekly; Th1: T-helper 1 cells; T-helper 2 cells; IL -4: Interleukin-4; IL-10: Interleukin-10.

**Table 4:** Animal Studies Examining Alternative Glatiramer Acetate Outcomes in Multiple Sclerosis.

Disease Model	Research Model and Methodology	Findings	Ref.
**MS**	6–8-week-old m&f SJL/L mice (n = 8 mice/group) EAE, MOG 33–55 peptide (MS model) [106] GA-immunized, q2d (Or treated with EGCG 300 μg/oral/q2d) PBS-injected or naïve wild type	IHC and EM: improved neuronal survival, axonal growth, remyelination, formation of new synapses and axonal regeneration ELISA: increased BDNF LSS: improved motor and cognitive functioning Improved neurogenesis, reduced disease progression and higher BDNF levels.	Herges, K. et al., 2011 [102]
**MS**	8–10-week-old m&f, C57BL/6 mice GA-treated (n = 27) PBS-injected (n = 22) or naïve wild type (n = 24)	CMT, GA vs. placebo and WT: higher levels of STM LSS, GA vs. placebo: No decline appreciated or a slower rate of decline IHC and EM, GA vs. placebo: reduced cortical damage Improved STM/cognition and less memory decline (LSS and CMT).	LoPresti, P. 2015 [107]
**MS**	8–12-week-old f C57BL/6 mice GA-immunized (n = 12) PBS-injected (n = 12) or naïve wild type (n = 10)	Improved short-term memory, reduced mistakes in CMT IHC and EM [mean ± SD], GA vs. placebo: astrocyte processes overlap barrel boundaries [13.1 ± 0.5] vs. [5.8 ± 0.3] (*p* < 0.001) GM-CSF [108] Clasping score GA vs. placebo: less GM-SCF expressing cells 20% of T-cells (*p* < 0.01), 72% of macrophages (*p* < 0.05), 31% of leukocytes (*p* < 0.0001) Reduced cognitive decline (LSS and CMT) and improved astrocyte morphology/vascular connections	Eilam, R. et al., 2018 [109]
**MS**	5–8-week-old m&f SJL/L mice GA-immunized, 50 μg/s.c./q2d (n = 13) PBS-injected (n = 12) or naïve wild type (n = 10)	DNMSTM GA vs. placebo: χ^2^_(4)_ = 7.506 (*p* = 0.111) IHC and EM GA vs. placebo: smaller, lower number of cellular infiltrations and moderate/absent astrocyte and microglial activation Preserved cognitive function and provided neuroprotection against cellular invasion/inflammation	Aharoni, R. et al., 2019 [110]
**MS**	8–10-week-old f mice GA-immunized (n = 22), ABAH treated (n = 19), or combo treatment (n = 31) PBS-injected controls (n = 22)	GA and combo treatment vs. placebo Disease onset, [mean # of days]: [10.4] and [11.3] vs. [9.0] (*p* < 0.05) Disease severity, GA and GA-combo treatment vs. placebo [mean]: [3.1] and [1.8] vs. [3.9] (*p* < 0.05) MPO^+^ lesions GA and combo treatment vs. placebo [mean]: [64.8] and [30.2] vs. [67.2] (*p* < 0.05) Reduced inflammatory plaques #/activity/size (monitored w/MPO on Gd-MRI). Improved cognition (LSS scores).	Li, A. et al., 2019 [111]

EAE: Experimental Autoimmune Encephalomyelitis; EAE model used to induce MS-like state in all studies. Test subjects were administered 200–250 μg/s.c./qd, unless otherwise specified; MS: multiple sclerosis; m&f: male and female; MOG: myelin oligodendrocyte protein; s.c.: subcutaneous; q2d: every 2 days; EGCG: Epigallocatechin 3-Gallate; LSS: Longa scoring scale; IHC: immunohistochemistry; EM: electron microscopy; ELISA: enzyme-linked immunosorbent assay; BDNF: brain-derived neurotrophic factor; qd: daily; CMT: cross-maze test; GM-CSF: granulocyte–macrophage colony-stimulating factor; DNMSTM: delayed non-matching to sample T-maze; ABAH: 4-aminobenzoic acid hydrazide; MRI: magnetic resonance imaging; MPO: Myeloperoxidase; Gd^+^: gadolinium positive.

**Table 5:** Animal Studies Examining Alternative Uses of Glatiramer Acetate in Amyotrophic Lateral Sclerosis.

Disease Model	Research Model and Methodology	Findings	Ref.
**ALS**	10–12-week-old f B6SJ/L mice Overexpression of G93A-SOD1 gene (ALS model) GA-immunized (n = 14) PBS-injected (n = 13) and naïve wild type (n = 12)	Lifespan GA vs. control [mean days ± SD]: [211 ± 7] vs. [263 ± 8] Higher levels of motor neurons after facial nerve axotomy, compared to controls (*p* < 0.05) Improved/protected motor activity via biometrically analyzed whisking behavior Increased life expectancy, motor number, and improved motor activity/function.	Angelov, D. et al., 2003 [99]
**ALS**	Male tg B6SJL-tg (SOD1-G93A)1Gur mice crossbred with female non-tg B6SJLF1-mice; offspring tested at 40 days old (n = 9 mice/group) GA-immunized vs. PBS-injected controls	RAWM GA vs. placebo: Delayed impairment of motor function and lessened disease progression GA vs. placebo: reached 10% of pre-symptomatic functional activity Motor function improved/protected. Disease progression slowed	Habisch, H. et al., 2007 [116]
**ALS**	50-day-old m&f B6SJL-tg [SOD1-G93]1Gur mice; B6. cg-tg [SOD1-G93A]1Gur/J mice; SOD1 G37R mice (n = 15–17 mice/group) TV-5010, 75,200 or 500 μg/s.c. qw, q2w or monthly GA-immunized vs. PBS-injected controls	Muscle strength (disease onset): no significant change No significant changes in lifespan (delayed lifespan phenotype) Significant diminution of survival for mice treated qw compared to other treatment regimens (*p* < 0.05) Rotarod: no significant improvements/changes in motor function Regimens had minor differences in findings Study utilized TV-5010 (synthetic HMW polymer formulation of the same amino acids of GA).	Haenggeli, C. et al., 2007 [117]

Animals in the treatment groups received 100 μg/s.c./qw unless otherwise specified; ALS: amyotrophic lateral sclerosis; SOD1: superoxide dismutase 1; tg: transgenic; RWA: running wheel activity; HMW: high molecular weight.

**Table 6:** Animal Studies Examining Alternative Uses of Glatiramer Acetate in Alzheimer’s Disease.

Disease Model	Research Model and Methodology	GA Effects/Findings	Ref.
**AD**	10–12-week-old m&f APP_SWE_/PS1_ΔE9_ mice * and non-Tg WT littermates GA-immunized (n = 5) PBS-injected (n = 8) and naïve WT (n = 7)	Aβ fibrils: 70% reduction (*p* < 0.02) Aβ fibrils in hippocampus: 92% reduced (*p* < 0.01) 31% reduction in astrocytosis (*p* = 0.039) GA-enhanced microglial activation correlated w/decreased Aβ fibrils.	Frenkel, D. et al., 2005 [125]
**AD**	8–10-month-old m&f APP_SWE_/PS1_ΔE9_ mice ^@^ and non-Tg WT littermates GA-immunized vs. PBS-injected and naïve WT (n = 7–8 mice/group)	GA enhanced protective microglia (CD11b^+^/CD11c^+^/MHC class II^+^/TNF-α^−^) Eliminated Aβ plaque formation (*p* < 0.05) MWMT: GA learning and memory improved (*p* < 0.0001) Reduced cognitive decline (MWMT) and increased neurogenesis.	Butovsky, O. et al., 2006 [18]
**AD**	3-month-old m&f APP_SWE_/PS1_ΔE9_ (ADtg)-CD11c*^DTR–GFP^* chimeric mice ^#^ GA-immunized vs. GA-immunized with DT, vs. untreated ADtg chimeric mice Nonchimeric ADtg mice controls: GA-immunized vs. untreated (n = 3–4 mice/group)	Reduced CD11^+^ proinflammatory cells Promoted/enhanced neuroprotection and neurogenesis Enhanced removal of Aβ-plaque Lessened Aβ plaque formation and provided neuroprotection	Butovsky, O. et al., 2007 [128]
**AD**	7-month-old m&f APP_SWE_/PS1_ΔE9_ mice ^@^ and non-Tg WT littermates Weekly GA or PBS for 12 weeks (n = 7 mice per group) and naïve WT	GA vs. controls, mice and rats Scar tissue: 8% vs. 15% Protein degradation/ubiquitination: 0% vs. 6% Growth/neurogenesis: 13% vs. 9% Development/migration/differentiation: 115% vs. 8% Transcription regulation: 14% vs. 5% 35% increase in hippocampal EGR1 (*p* < 0.01) Enhanced neurogenesis in hippocampus Induced neurogenesis, neuroplasticity and neuroprotective gene activation-*Egr1* likely to be involved in GA-mediated enhanced capacity for regeneration in the DG and improved cognition.	Bakalash, S. et al., 2011 [95]
**AD**	10-month-old m APP_swe_/PS1_∆E9_ mice ^@^ and non-Tg WT littermates Weekly GA or PBS vs. GA-plus CD115^+^ Mo^BM^ adoptive transfer ** vs. and naïve WT for 8 weeks in 10-month-old (n = 6–8 per group)	GA vs. controls Aβ levels reduced (*p* < 0.001) and astrogliosis reduced (*p* < 0.0001) Enhanced monocyte recruitment—associated w/IL-10 driven phagocytosis of Aβ plaques Increased MMP9 protein (*p* < 0.05) Enhanced macrophage-phagocytosis of fibrillar Aβ_42_ (*p* < 0.0001) Significant plaque reductions, 40–53% (hippocampus) and 61–78% (cortex) (*p* < 0.0001–0.001) Improved BMT scores (*p* < 0.001) Synaptic preservation Enhanced Aβ degradation, attenuated disease progression, improved memory and learning	Koronyo, Y. et al., 2015 [32]
**AD**	4–7-month-old 5xFAD mice ^ and non-Tg WT littermates Four conditions: (a) Weekly GA for 1 or 4 weeks in 4-month-old mice; (b) Weekly GA for 4 weeks in 5-month-old mice; (c) Twice a week GA for 1 week in 6-month-old mice; (d) Daily vs. weekly for 4 weeks in 7-month-old mice GA-immunized vs. PBS-injected 5xFAD mice and naïve WT mice (n = 4–8 per group)	Enhanced expression of BDNF and IGF-1; increased IFN-γ RAWM: improved spatial memory Reduced neuroinflammation and Foxp3^+^ Treg levels Weekly GA injections reversed Aβ plaque formation and improved RAWM cognitive performance Daily GA injections led to moderately worsened cognition (RAWM results) and no clearance of Aβ plaques Weekly GA improved cognition (spatial memory), reduced neuro and peripheral inflammation, and decreased Aβ plaque burden	Baruch et al., 2015 [129]
**AD**	10-month-old m APP_SWE_/PS1_ΔE9_ mice ^@^ and WT littermates In vivo: (a) Weekly GA or PBS vs. GA- plus CD115^+^ Mo^BM^ adoptive transfer ** for 8 weeks in 10-month-old mice (n = 4–6 per group); (b) Weekly GA for 4 weeks in 3-month-old mice (n = 3 per group) In vitro: WT MΦ^BM^ CD115^+^ treated with 30 μg/mL GA for 24 h (n = 3–5 replicates)	GA vs. controls Increased OPN-expressing MΦ Enhanced Aβ phagocytosis Reduced Aβ cerebral and vascular pathology GA increased OPN and MΦ^BM^, 1.4–2.5 times higher than controls (*p* < 0.01–0.0001) Enhanced OPN expression and reduced Aβ plaques	Rentsendorj, A. et al., 2018 [127]
**AD**	20-month-old m&f APP_SWE_/PS1_ΔE9_ mice ^@^ and wild type littermate mice Weekly GA or PBS for 8 weeks and naïve WT (n = 6–7 mice per group)	GA vs. controls Diminished vascular and parenchymal Aβ deposition Restoration of post-synaptic biomarker PSD-95 density Reduced Aβ_42/40_ ratio levels in retina (*p* = 0.0246) 63% reduction in vascular amyloidosis (*p* = 0.0093) Reduced microgliosis and reactive astrocytosis (*p* = 0.0361) Increased cerebral infiltrating CD45^hi^/Iba1^+^ monocyte-derived macrophages (*p* < 0.001–0.0001) Restored homeostatic astrocyte phenotype (i.e., GFAP, GS expression) (*p* = 0.005) Aβ-plaque reduction—brain regions and plaque subtypes: Hippocampus: 40% reduction (*p* = 0.0003) Cortex: 48% reduction (*p* = 0.0001) Total brain: 46% reduction (*p* = 0.0001) Large, hard-core plaques: 28% reduction (*p* = 0.0017) Synaptic preservation Correlation and similar reduction in retinal and brain Aβ plaques; tissue homeostasis and regeneration	Doustar, J. et al., 2020 [105]
**AD**	10-month-old m APP_SWE_/PS1_ΔE9_ mice ^@^ and WT littermates In vivo: Weekly GA or PBS vs. CD115^+^—MΦ^BM^ adoptive transfer ** for 8 weeks and naïve WT mice (n = 6 mice/group) In vitro: WT MΦ^BM^ treated with 30 μg/mL GA for 1, 3, or 24 h (n = 3–4 replicates)	GA vs. controls GA-induced MΦ^BM^ phagocytosed f/oAβ_42_ fibrils BMT: Improved cognitive function 36% decrease in Aβ_42_ of GA-macrophages (*p* < 0.01) Synaptic preservation Increased levels of protective MΦ^BM^ and enhanced cognition	Li, S. et al., 2020 [124]
**AD**	15-month-old f 3xTg mice ^$^ and non-Tg mice Weekly GA or PBS for 8 weeks, 500 ng/μL and naïve wild type (n = 9–11 mice per group)	Increased discrimination index (novel object recognition vs. former object) over 8 weeks (*p* = 0.01) and significant difference from placebos (*p* = 0.04) IHC: decrease in hippocampal Aβ_1–42_ after 8 weeks of GA use (*p* = 0.02) Improved cognition, reduced amyloid plaque deposition	Dionisio-Santos, D. et al., 2021 [126]

All studies utilized transgenic models of AD. Animals in the treatment groups received 100 μg/s.c./qw, unless otherwise specified; AD: Alzheimer’s disease; Aβ: Aβ; MWMT: Morris water maze test; RAWM: radical arm water maze; SGZ: subgranular zone; m: male; qm: monthly; BMT: Barnes maze test; MΦ^BM^: bone-marrow-derived monocytes/macrophages; DG: dentate gyrus; MEA: multi-electrode analysis; f/oAβ_42_: fibrillar/oligomeric Aβ_42_; IOP: intraocular pressure; RT-PCR: real time-polymerase chain reaction; WB: Western blot; EGR1: early growth response gene 1; MMP9: matrix metallopeptidase 9; FAD—familial Alzheimer’s disease; DT—diphtheria toxin. ** CD115^+^ Mo^BM^: Adoptive transfer of CD115^+^ bone-marrow-derived monocytes isolated from 8- to 10-week-old GFP-labelled C57BL/6 mice. MΦ^BM^: bone-marrow-derived monocytes/macrophages isolated from 8- to 10-week-old C57BL/6 mice injected monthly for 2 months. Murine models (listed age is at the start of the experiment): * Double-transgenic amyloid precursor protein (*APP*) barring the Swedish FAD mutations (K595N, M596L) + presenilin 1 (*PS1*) with deletion in exon 9 mice, called APP_SWE_/PS1_ΔE9_ on C57/BL6-SJL background. ^@^ The APP_SWE_/PS1_ΔE9_ on C57BL/6 background [B6.Cg-Tg (APPswe, PSEN1dE9) 85Dbo/J mouse strain]. ^#^ Chimeric APP_SWE_/PS1_ΔE9_ on C57BL/6 background after lethal whole-body irradiation and reconstitution with 5 × 10^6^ bone marrow cells isolated from 2-month-old C57BL/6 J-CD11c*^DTR–GFP^* mice. The latter is a transgenic CD11c*^DTR–GFP^* mouse, carrying a transgene encoding a human diphtheria toxin receptor (DTR)–green fluorescent protein (GFP) fusion protein under control of the murine CD11c promoter [130]. ^ Heterozygous 5XFAD transgenic mice (Tg6799; on a C57/BL6-SJL background) co-overexpressing FAD mutant forms of human *APP* (the Swedish mutation, K670N/M671L; the Florida mutation, I716V; and the London mutation, V717I) and mutant *PS1* (M146L/L286V) transgenes under control of the neuron-specific mouse Thy-1 promoter. ^$^ 3xTg AD mice express mutated human *APP* Swedish, *MAPT* P301L, and *PSEN1* M146V genes under transcriptional control of the neuron-specific mouse Thy1.2 promoter [131]. Control mice: Wild type (WT) non-transgenic (Tg) littermates.

**Table 7:** Animal Studies Examining Alternative Uses of Glatiramer Acetate in Parkinson’s Disease.

Disease Model	Research Model and Methodology	Findings	Ref.
**PD**	7–10-week-old m&f C57BL/6 mice MTPT (neurotoxin PD model) MTPT mice—injected w/ serum from mice immunized with GA 200 μg/s.c. weekly PBS-injected controls (n = 14)	TH^+^-neuron levels correlated to immune cell number (regression analysis, r = 0.96) Protected SN from MPTP-induced neurodegeneration Enhanced anti-inflammation cytokine proliferation and BDNF/GDNF Inhibited dopaminergic neurodegeneration Improved density of dopaminergic striatal termini Reduced disease progression, increased BDNF/GDNF, IL-4 and IL-10	Laurie, C. et al., 2007 [137]
**PD**	8-week-old m&f C57BL/65 MPTP mice GA 3.5 mg/kg/s.c./daily (n = 25) PBS-injected (n = 30) and naïve wild type (n = 24)	GA vs. controls Diggigait test: improvement/reversal of motor dysfunction TH: smaller decrease 16% (*p* = 0.1953) 51% increase in grip strength (F(5,90) = 63.38, *p* < 0.0001) Brake time was restored, equal to healthy controls (*p* = 0.0439) Higher levels of TH linked to enhanced cognition and motor activities	Churchill, M. et al., 2019 [135]

All studies utilized the MPTP PD model; MPTP: 1-Methyl-1,2,3,6-Tetrahydropyridine; TH: Tyrosine Hydroxylase; PD: Parkinson Disease; SN: Substantia Nigra.

**Table 8:** Animal Studies Examining Alternative Uses of Glatiramer Acetate in Huntington’s Disease.

Disease Model	Research Model and Methodology	Findings	Ref.
**HD**	10–12-week-old m N171-82Q and f C3B6F1 mice (n = 6–7 mice/group) Induced CAG repeat GA-immunized, 750 μg/s.c./qd PBS-injected and naïve wild type	GA vs. controls OFBA decreased hyperactivity and stereotypic behavior (F(1,110) = 8.81; *p* = 0.01) Elevated BDNF in striatal cells: [2.48 pg/mg] vs. [0.90 pg/mg] (*p* = 0.003) Prevented onset of motor deficits and cognitive issues—particularly if treatment began early in disease process	Corey-Bloom, C. et al., 2014 [142]
**HD**	10-week-old m&f B6CBA, C57BL/6, FVB and YAC128 mice (n = 4–10 mice/group) GA-immunized, 100 μg/s.c./qw vs. PBS-injected	GA vs. controls Increased average lifespan; increased levels of active BDNF Rotarod and Clasping Score [143]: improved motor performance OFBA: improved cognitive behaviors Preservation of damaged motor neurons Lengthened lifespan, improved cognition and motor function	Reick, C. et al., 2016 [140]
**HD**	1-year-old m&f CAG140 rats and 7-month-old m&f N171-82Q mice HD rats (n = 18), GA 100 μg/s.c./q5w and GA 625 μg/s.c./q3w PBS-injected, (n = 30)	GA vs. controls ATM [144]: Jump time (*p* = 0.029; F(1,150) = 4.8) OFBA and Rotarod: Less stereotypic time (F(1,150) = 16.5; *p* < 0.0001) Climbing test [145]/Grip test [146]: Resting time improved (F(1,150) = 9.0; *p* = 0.003) Delayed disease onset and improved lifespan Elevated BDNF and decreased proinflammatory cytokines Improved stereotyped behavior, reduced behavioral issues, delayed disease onset and prolonged lifespan	Corey-Bloom, J. et al., 2017 [139]

All studies utilized the SOD1-induced HD model; HD: Huntington’s disease; OFBA: open field behavioral analysis; ATM: alternating T-maze.

**Table 9:** Animal Studies Examining Alternative Uses of Glatiramer Acetate in Neuropsychology.

Disease Model	Research Model and Methodology	Findings	Ref.
**Psych**	8–12-week-old m C57BL/6J and BALB/c/OLA mice RAG ½ knockout/nude mice (SCID model) [149] MK-80 [150] and AMPH [151] GA-immunized, qd, (n = 6) vs. PBS-injected (n = 7)	Less cognitive impairment linked to psychometric agents (MK-80 and AMPH) PPI [152]: Enhanced communicative behavior MWMT: Sensorimotor dysfunction was prevented Enhanced cognition and behavior, improved impairments induced by psychomimetic agents	Kipnis, J. et al., 2004 [103]
**Neuro** **psych**	6-week-old m Sprague–Dawley rats (n = 7 rats/group) Cranially irradiated [153] GA-immunized, qw PBS-injected and naïve non-irradiated rats	MWMT: Reversal of behavior impairment; better cognitive abilities; shorter latency times (*p* < 0.01) Restored hippocampal neurogenesis Increased BDNF, IGF-1, and IFN-γ levels; decreased TNF-α, IL-6, and IL-4 levels Reversal of cognitive deficits, enhanced GA-mediated/immune-induced hippocampal neurogenesis and increased protective cytokines	He, F. et al., 2014 [154]
**Neuro** **psych**	12–16-week-old f BALB/c mice (n = 25 mice/group) CMS exposure [155] GA-immunized, qw PBS-injected CMS and non-CMS	OFBA and OIPT [156] GA vs. control Reversed effects of CMS on learning and memory (*p* < 0.0001) Regulated hipp. NOS activity/reduction in ROS Number of crossings: (t(18) = 4.461, *p* < 0.001) Rearing: (t(18) = 7.313, *p* < 0.001) Corner time: (t(18) = 3.478, *p* < 0.001) Improved cognition and neuronal functioning and repaired cortical damage	Pascuan, G. et al., 2015 [157]
**Neuro** **psych**	6–8-week-old m C57BL/6 mice (n = 6–10 mice/group) LPS induction [158] (memory impairment model) GA-immunized, 250 μg/s.c./qw vs. PBS-injected	GA vs. control YMT [159] and PAT [160]: Less time exploring maze arms; [F(2,20) = 7.407], (*p* < 0.01, [F(2, 20) = 10.433]) Increased novel arm time; improved spatial recognition and memory Shock fear memory: Shorter latency times (*p* < 0.01) Improved retention trials [F(1,11) = 16.773; *p* < 0.001] Neuroprotective effects were notably seen in a dose-dependent manner	Mohammadi, F. et al., 2016 [161]

Test subjects in the treatment group received 100 μg/s.c., unless otherwise specified; Neuropsych: Neuropsychology; Psych: Psychology; SCID: severe combined immunodeficiency; MK-80: dizocilpine maleate; AMPH: d-amphetamine sulfate; PPI: pre-pulse inhibition; MWMT: Morris water maze test; CMS: chronic mild stress; OFBA: open field behavioral analysis; OIPT: object in place test; NOS: nitrous oxide; ROS: reactive oxygen species; LPS: lipopolysaccharide; YMT: Y-maze test; PAT: passive avoidance task.

**Table 10:** Animal Studies Examining Alternative Uses of Glatiramer Acetate in Vascular Dementia and Central Nervous System Ischemia.

Disease Model	Research Model and Methodology	Findings	Ref.
**CNSi**	12-week-old m Sprague–Dawley rats tMCAo (CNSi model) [166] GA-immunized (n = 6) PBS-injected (n = 6)	LSS: Improvement in neurological function (1.2 ± 0.4 and 2.8 ± 0.5; *p* = 0.008) Higher tissue preservation Reduced infarct volume (4.8 ± 1.5), vs. controls (32.2 ± 8.6; *p* = 0.004)Neuroprotective effects; improvements in neurocognition and infarct volume	Ibarra, A. et al., 2007 [164]
**CNSi**	10-week-old m Lewis rats Chronic cerebral hypoperfusion (VD model) GA-immunized, 100 μg/s.c./qw (n = 8) PBS-injected (n = 8)	MWMT, GA vs. control Shorter latency swim times (*p* < 0.01) More time in novel maze areas (*p* < 0.5) Higher number of platform crossings (*p* < 0.01) Reduced # of GFAP^+^ cells in hippocampus (*p* < 0.01) Less IFN-γ, IL-6, and TNF-α (*p* < 0.05, *p* < 0.01, *p* < 0.01) Increased BDNF in hippocampus (*p* < 0.01) Reduced pathology changes and attenuated cell loss Restored brain’s immune microenvironment Restored cognitive and neuronal functioning; slowed disease progression	Chen, L. et al., 2015 [167]
**CNSi**	7-week-old Sprague–Dawley m rats (n = 4–8 rats/group) GA-immunized vs. PBS-injected controls	GA vs. control [mean ± SD] LSS: [1.0 ± 0.8] vs. [1.9 ± 0.6] (*p* < 0.01) Infarcts’ volume: [8.9 ± 1.9] vs. [18.5 ± 1.1%] (*p* < 0.05) Cognitive function recovery time: [0.5 ± 0.5] vs. [1.4 ± 0.5] (*p* < 0.01) Neurogenesis, ipsilateral SVZ: [260 ± 86] vs. [155 ± 61] (*p* < 0.05) Neurogenesis, contralateral SVZ: (170 ± 63 vs. 107 ± 53; *p* < 0.05) Enhanced neuroprotective/neural progenitor cells in SVZ, SGZ, and cortex Enhanced neurogenesis and decreased infarct volume. Improved neurogenesis, less cognitive decline, reduced infarct volume, accelerated movement recovery	Cruz, Y. et al., 2015 [168]
**CNSi**	5-week-old m Sprague–Dawley rats GA-immunized (n = 6) PBS-injected (n = 7) and naïve non-tMCAo (n = 6)	GA vs. control LSS: Reduction in neuro deficit (*p* < 0.001) Upregulated BDNF, IGF-1, and IL-10; downregulation of IL-17 Increase neuroblasts, SVZ (*p* < 0.0001) and neurogenesis, SVZ/SGZ Increased neuroblasts, SVZ—negative correlation w/neuro deficits (*r* = −0.86, *p* < 0.05) Neurogenesis SVZ, reduced neuro deficits (*r* = 0.86, *p* < 0.05) Ameliorated neuro deficits, more neurogenesis and increased BDNF	Cruz, Y. et al., 2018 [169]
**CNSi**	6-week-old m C57BL/6J mice Induction of diabetes and cerebral ischemia by pMCAo [170] GA-immunized, q3d (n = 16) vs. PBS-injected (n = 17)	GA vs. control Normalized neuro scores in sensorimotor domains (*p* = 0.0018) Increased BMT scores (*p* < 0.01) Retention task [171] was improved Grip test/beam walking [172] better long-term spatial memory BMT and Pole test [173]: increased latency (*p* < 0.05) Reduced infarct volume by 40% [11.78 ± 1.60 mm^3^] (*p* = 0.016) Less proinflammatory mediators: COX2, CD32, TNFα, and IL-1β Reduced infarct volume, little/no cognitive impairments or long-term deficits	Mangin, G. et al., 2019 [174]

Test subjects in the treatment group received 200 μg/s.c./qw, unless otherwise specified; VD: vascular dementia; CNSi: central nervous system ischemia; pMCAo: permanent middle cerebral artery occlusion; q3d: every 3 days; SVZ: subventricular zone; SGZ: subgranular zone; DG: dentate gyrus; tMCAo: temporary middle cerebral artery occlusion.
